# MRI-Based Knee Morphometry and Pediatric Anterior Cruciate Ligament Injury: A Machine Learning Case–Control Study

**DOI:** 10.3390/jcm15187246

**Published:** 2026-09-17

**Authors:** Murat Onder, Anil Erbas, Muhammet Alptekin Kocaoglu, Mert Colak, Muhammet Mert, Muhammed Bilal Kurk, Abdulhamit Misir

**Affiliations:** 1 Department of Orthopaedics and Traumatology, Medicana Zincirlikuyu Hospital, Esentepe Mahallesi, Büyükdere Caddesi No:165, 34394 İstanbul, Türkiye; 2 Department of Orthopaedics and Traumatology, Baltalimani Bone Diseases Training and Research Hospital, 34470 İstanbul, Türkiye; 3 Department of Orthopaedics and Traumatology, Central Hospital Kozyatağı, 34742 İstanbul, Türkiye; 4 Department of Orthopaedics and Traumatology, Medical Park Maltepe Hospital, 34846 İstanbul, Türkiye; 5 Department of Orthopedics and Traumatology, Istanbul Aydin University Faculty of Medicine, 34295 İstanbul, Türkiye

**Keywords:** anterior cruciate ligament, pediatric, magnetic resonance imaging, knee morphometry, machine learning, feature selection

## Abstract

**Background/Objectives**: Anterior cruciate ligament (ACL) injury is an important cause of knee instability and long-term morbidity in children and adolescents. Although several anatomical characteristics of the knee have been associated with ACL injury, these features are commonly evaluated in isolation despite the multidimensional nature of knee morphology. This study aimed to characterize MRI-based morphometric differences associated with pediatric ACL injury and to determine whether multidimensional knee morphology could discriminate ACL-injured from control knees using machine-learning approaches. **Methods**: This retrospective, single-center, case–control study included 462 patients 18 years of age or younger who underwent knee magnetic resonance imaging (MRI) between January 2012 and June 2025. The cohort comprised 233 patients with ACL injury and 229 controls with an intact ACL. Eighteen MRI-derived femoral and tibial morphometric parameters were evaluated. Six feature-selection methods were combined with six classification algorithms, resulting in 36 model combinations assessed using repeated 10-fold cross-validation. Model discrimination, calibration, and feature-selection stability were evaluated. Feature selection was performed independently within each training fold to minimize information leakage. **Results**: In total, 13 of the 18 morphometric parameters differed significantly between groups after false discovery rate correction, including medial and lateral tibial slopes, medial tibial depth, lateral femoral condyle ratio, and intercondylar notch angles. Mean area under the receiver operating characteristic curve (AUC) values across the 36 model combinations ranged from 0.908 to 0.959. least absolute shrinkage and selection operator (LASSO) combined with naive Bayes achieved the highest mean AUC (0.959; 95% CI, 0.905–0.992), although 18 combinations showed statistically comparable discrimination. LASSO combined with logistic regression demonstrated similarly high discrimination (AUC, 0.957) with favorable calibration (Brier score, 0.080; calibration slope, 0.91; intercept, 0.01). LASSO also demonstrated the highest feature-selection stability (Nogueira index, 0.985; mean Jaccard similarity, 0.986), consistently retaining eight core features across all resampling iterations. **Conclusions**: Pediatric ACL injury was associated with a distinct multidimensional MRI-based morphometric profile involving both tibial and femoral anatomy. Multiple machine-learning approaches demonstrated high discrimination between ACL-injured and control knees, while LASSO-based feature selection showed particularly high reproducibility. These findings support the presence of a stable morphometric signature associated with pediatric ACL injury. The models should be interpreted as classifiers of established ACL injury status rather than prospective predictors of future injury.

## 1. Introduction

Anterior cruciate ligament (ACL) injury is a major cause of knee instability and functional impairment in children and adolescents, and its clinical relevance has increased in parallel with the growing participation of younger individuals in organized and competitive sports [1]. Epidemiological studies have demonstrated increasing rates of ACL injury and reconstruction in pediatric and adolescent populations, with particularly high injury rates among athletes participating in pivoting and cutting sports [2,3]. Although contemporary treatment strategies allow a substantial proportion of pediatric patients to return to sport, ACL injury at a young age remains associated with important short- and long-term consequences, including secondary meniscal and chondral damage, recurrent ipsilateral injury, and contralateral ACL injury [4,5]. These consequences, together with the unique considerations associated with skeletal immaturity, have increased interest in identifying intrinsic factors that may contribute to ACL injury susceptibility in the developing knee [6].

ACL injury susceptibility is multifactorial and reflects interactions among demographic, biomechanical, neuromuscular, and anatomical characteristics. Among potentially measurable intrinsic factors, osseous morphology of the tibiofemoral joint is particularly relevant because it can be objectively quantified using magnetic resonance imaging (MRI). Previous investigations have associated ACL injury with increased posterior tibial slope and altered medial tibial plateau depth [7,8], while pediatric MRI-based studies have demonstrated associations involving lateral tibial slope and intercondylar notch morphology [9,10]. Other investigations have further implicated intercondylar notch angle and related femoral morphological characteristics in ACL injury susceptibility [11]. However, these anatomical parameters are not independent structures; rather, they collectively define the geometry of the tibiofemoral joint and may interact in ways that cannot be fully characterized by evaluating individual measurements in isolation. Machine-learning approaches provide an opportunity to integrate multiple correlated morphometric features, model potentially complex relationships, and evaluate which combinations of anatomical characteristics most effectively discriminate ACL-injured from uninjured knees. Recent studies have demonstrated the feasibility of applying machine learning to MRI-derived knee morphology, and a recent case–control study showed that machine-learning models incorporating tibial anatomical variables could effectively discriminate individuals with and without ACL injury [12]. Nevertheless, comprehensive machine-learning analyses integrating a broad range of femoral and tibial MRI-based morphometric characteristics specifically in pediatric patients remain limited.

Accordingly, the contribution of the present study lies not in the application of machine learning per se, but in the pediatric-specific integration of a relatively broad set of femoral and tibial MRI-derived morphometric characteristics, systematic comparison of multiple feature-selection and classification strategies, and assessment of feature-selection stability within a common analytical framework. This study aims to investigate MRI-based knee morphometric characteristics associated with ACL injury in a pediatric population and to determine whether multidimensional anatomical information can discriminate between ACL-injured patients and uninjured controls using machine-learning approaches. Specifically, 18 femoral and tibial morphometric parameters were evaluated using multiple feature-selection strategies combined with different classification algorithms. In addition to discrimination performance, model calibration and feature-selection stability were assessed to provide a comprehensive evaluation of model reliability and the reproducibility of the identified anatomical features. We hypothesized that pediatric ACL injury is associated with a distinct knee morphometric profile and that machine-learning models integrating these anatomical characteristics would demonstrate robust discrimination between ACL-injured and uninjured knees.

## 2. Materials and Methods

### 2.1. Study Design and Ethical Approval

This retrospective, single-center, case–control study was conducted at Metin Sabancı Baltalimanı Bone Diseases Training and Research Hospital. The institutional imaging archive was retrospectively reviewed to identify pediatric patients who underwent knee MRI between January 2012 and June 2025. The study protocol was approved by the Scientific Research Ethics Committee of Metin Sabancı Baltalimanı Bone Diseases Training and Research Hospital (approval date: 30 April 2026; decision no. 2/14) and was conducted in accordance with the principles of the Declaration of Helsinki. Given the retrospective nature of the study and the use of previously acquired clinical imaging data, the requirement for individual informed consent was waived by the institutional ethics committee. All study-specific data extraction, morphometric measurements, and statistical analyses were initiated only after institutional ethics committee approval had been obtained. Demographic characteristics recorded for each participant included age, sex, and laterality of the examined knee.

### 2.2. Study Population and Eligibility Criteria

Patients were eligible for the ACL-injured group if they were 18 years of age or younger, had an ACL injury confirmed on knee MRI, and had MRI examinations of sufficient diagnostic quality to permit reliable evaluation of ACL integrity and completion of the predefined morphometric measurements. Both partial and complete ACL tears were eligible for inclusion, and patients were not stratified according to acute or chronic injury status. Patients with previous ACL reconstruction or prior ipsilateral knee surgery were excluded because prior surgical intervention could alter the native anatomy relevant to the morphometric measurements. Exclusion criteria were age >18 years, equivocal assessment of ACL integrity on MRI, congenital abnormalities of the knee or skeletal dysplasia, fractures around the knee that could alter the anatomical measurements, and multiligamentous knee injuries. The control group consisted of patients 18 years of age or younger who underwent knee MRI during the same study period for clinical indications but demonstrated an intact ACL. Controls were required to have MRI examinations of sufficient quality to permit completion of all predefined morphometric measurements. The most common indications for MRI in the control group were nonspecific knee pain (34.9%), persistent pain or sprain following sports activity or minor trauma (27.1%), suspected meniscal pathology (19.7%), patellofemoral pain or suspected patellar instability (13.1%), and other indications (5.2%). The indication for MRI referral was distinguished from the final MRI findings: for example, patients referred for suspected meniscal pathology in whom no meniscal tear was identified on MRI remained eligible for inclusion in the control group. Conversely, patients with an MRI-confirmed meniscal tear, a clinically significant chondral or osteochondral lesion, patellar dislocation or objective patellar instability, another major ligament injury, prior ipsilateral knee surgery, congenital or developmental abnormalities, or fractures affecting knee morphology were excluded from the control group. Only minor incidental findings not expected to affect the tibiofemoral morphometric measurements evaluated in this study were permitted. Accordingly, the control group should be regarded not as a cohort of healthy volunteers but as clinical controls who underwent MRI for a clinical indication, had an intact ACL, and had no significant structural knee pathology expected to affect the morphometric parameters under investigation. The same imaging eligibility criteria and morphometric definitions were applied to cases and controls.

### 2.3. Outcome Definition and Candidate Imaging Features

The primary outcome was ACL injury status, defined according to MRI evidence of ACL disruption. For study-group assignment, ACL integrity was determined on the basis of MRI assessment using the predefined imaging criteria. Both partial and complete tears were classified within the ACL-injured group. Clinical examination and operative confirmation were not required for classification because the study was designed as an imaging-based retrospective case–control analysis. Participants were categorized into an ACL-injured group and a control group with an intact ACL, and this binary classification served as the target outcome for subsequent machine-learning analyses.

Eighteen predefined MRI-derived morphometric parameters constituted the candidate imaging features: intercondylar notch width, notch width index (NWI), lateral tibial slope (LTS), medial tibial slope (MTS), medial meniscus–cartilage angle, medial tibial depth, lateral femoral condyle anteroposterior distance, proximal tibial anteroposterior distance, tibial eminence width, tibial eminence width index, notch-to-eminence ratio, notch shape index (NSI), notch height, lateral femoral condyle ratio (LFCR), coronal intercondylar notch angle, axial intercondylar notch angle, axial lateral wall angle, and coronal lateral wall angle. Age, sex, and laterality were recorded for demographic characterization and assessment of baseline comparability between groups but were not included among the 18 morphometric candidate features used for machine-learning classification.

### 2.4. MRI Acquisition and Image Review

Knee MRI examinations were performed throughout the study period using the same 1.5-T MRI system (SIGNA Explorer, GE Healthcare, Chicago, IL, USA) with a dedicated knee coil according to the institutional routine knee MRI protocol. Multiplanar imaging included sagittal, coronal, and axial fluid-sensitive and proton-density-weighted sequences suitable for assessment of ACL integrity and osseous morphometry. The routine protocol comprised sagittal proton-density-weighted and fluid-sensitive sequences, coronal T1-weighted and fluid-sensitive sequences, and axial fluid-sensitive imaging. Images were acquired using conventional two-dimensional sequences with approximately 3–4 mm section thickness, consistent with routine clinical knee MRI acquisition on this platform. Images were retrieved and reviewed through the institutional ExtremePACS system (ExtremePACS, Ankara, Türkiye). MRI examinations and morphometric measurements were evaluated by two orthopedic surgeons, each with at least 5 years of clinical experience in the assessment of knee disorders. All measurements were performed using predefined anatomical landmarks and standardized measurement protocols. For each morphometric parameter, the mean of the measurements obtained by the two observers was used in the subsequent statistical and machine-learning analyses. Measurements were performed using the electronic distance and angle measurement tools available within the PACS environment. During morphometric assessment, clinical information and group allocation were concealed whenever feasible; however, because ACL disruption may be directly visible on MRI, complete imaging-based blinding to injury status could not be guaranteed. Measurements were therefore performed according to predefined anatomical criteria irrespective of ACL injury status.

### 2.5. MRI-Based Morphometric Measurements

All measurements were performed in the imaging plane providing optimal visualization of the relevant anatomical landmark. Linear measurements were recorded in millimeters, angular measurements in degrees, and normalized indices and ratios as dimensionless values. The same definitions, anatomical landmarks, and measurement procedures were applied to ACL-injured and control knees. To minimize potential observer bias during morphometric measurements, several precautions were implemented. Clinical history, prior radiology reports, and group assignment (ACL-injured vs. control) were withheld from both observers during measurement, and cases were evaluated in a randomized order that interleaved ACL-injured and control patients rather than being assessed in separate group-wise blocks. For each of the 18 parameters, a predefined anatomical landmark and the imaging plane in which it was best visualized were used, rather than a plane selected to display the ACL itself; most notch- and lateral-wall-related parameters were measured on coronal/axial images, whereas tibial slope, tibial depth, and lateral femoral condyle-related parameters required sagittal images. The two observers performed all measurements independently, without access to each other’s results. Because ACL disruption can be directly or indirectly apparent on MRI—including on the sagittal sequences used for some measurements—complete blinding to ACL status could not be guaranteed; this residual potential for observer bias is addressed further in the Limitations section. Full formulas, anatomical landmarks, and imaging planes for all 18 morphometric parameters are provided in Supplementary Table S1. Representative MRI examples illustrating the measurement technique for the five parameters identified as most influential in the machine-learning models are provided in Supplementary Figure S1.

#### 2.5.1. Tibial Morphometry

Medial and lateral tibial slopes were assessed separately on sagittal MRI. The longitudinal axis of the proximal tibia was established as the anatomical reference, and a line perpendicular to this axis was constructed. The medial tibial slope (MTS) and lateral tibial slope (LTS) were defined as the angles between this reference line and a tangent to the articular surface of the corresponding tibial plateau. The articular cartilage surface was used to define plateau inclination, consistent with MRI-based morphometric assessment previously applied in pediatric ACL populations [9].

Medial tibial depth was determined on the sagittal image demonstrating the greatest concavity of the medial tibial plateau. A line connecting the anterior and posterior margins of the articular surface was constructed, and the maximum perpendicular distance between this reference line and the deepest point of the medial plateau was recorded [8].

The proximal tibial anteroposterior distance was measured on the sagittal image demonstrating the maximal anteroposterior dimension of the proximal tibial plateau. This measurement was used to characterize overall proximal tibial size and to support interpretation of size-normalized morphometric parameters.

Tibial eminence morphology was characterized using both absolute and normalized measurements. Tibial eminence width was defined as the maximal transverse distance between the outer margins of the medial and lateral intercondylar eminences on the coronal image best demonstrating the tibial eminence. Tibial plateau width was defined, on the same coronal image, as the maximal distance between the outer cortical margins of the medial and lateral tibial plateaus. The tibial eminence width index (EWI) was calculated as tibial eminence width divided by tibial plateau width. The notch-to-eminence ratio was calculated as intercondylar notch width divided by tibial eminence width. The use of normalized indices was intended to reduce the influence of differences in overall knee dimensions within a growing pediatric population.

#### 2.5.2. Intercondylar Notch Morphometry

Intercondylar notch morphology was characterized using linear, ratio-based, and angular measurements. Intercondylar notch width was measured on the image demonstrating optimal visualization of the femoral condyles and intercondylar notch. The NWI was calculated as the ratio of intercondylar notch width to the corresponding bicondylar femoral width, thereby providing a size-normalized measure of notch dimensions. MRI-based assessment of notch dimensions and NWI has previously been used to investigate anatomical susceptibility to ACL injury, including in skeletally immature populations [10].

Notch height was measured from the superior aspect of the intercondylar notch to the reference line at the inferior extent of the femoral condyles. The NSI was calculated as intercondylar notch width divided by notch height and was used as a dimensionless descriptor of overall notch configuration.

The coronal intercondylar notch angle was measured on the coronal image providing optimal visualization of the notch by constructing lines along its medial and lateral walls and recording the angle formed by their intersection. The axial intercondylar notch angle was similarly determined on the axial image demonstrating the intercondylar region and posterior femoral condyles most clearly.

Lateral notch-wall orientation was additionally assessed in both axial and coronal planes. On axial images, a posterior condylar reference line was constructed connecting the most posterior cortical points of the two femoral condyles, and a second line was drawn tangent to the linear portion of the lateral intercondylar notch wall; the axial lateral wall angle was defined as the acute angle between these two lines. On coronal images, a distal femoral condylar reference line was constructed connecting the most inferior articular points of the medial and lateral femoral condyles, and a second line was drawn tangent to the lateral notch wall; the coronal lateral wall angle was defined as the angle between these two lines. These measurements were included to characterize aspects of intercondylar notch geometry that are not captured by width-based indices alone.

#### 2.5.3. Lateral Femoral Condyle Morphometry

The anteroposterior dimension of the lateral femoral condyle was measured on the sagittal image demonstrating the maximal condylar profile. Lateral femoral condylar morphology was further characterized using the LFCR, a size-normalized measure of posterior condylar geometry. The distal femoral longitudinal axis was established on sagittal images by fitting two best-fit circles to the distal femoral shaft and constructing a line through their centers. The midsagittal slice of the lateral femoral condyle was selected using the popliteal groove as a coronal reference. On this slice, the most anterior point (A) and most posterior point (B) of the lateral condyle were identified, and the intersection of the distal femoral longitudinal axis with line A–B was defined as point O. The LFCR was calculated as OB/AB, i.e., the ratio of the posterior condylar segment to the total anteroposterior condylar length. Increased LFCR has previously been associated with ACL injury [13], and subsequent studies have supported MRI-based assessment of this morphological characteristic [14].

#### 2.5.4. Medial Meniscus–Cartilage Morphometry

The medial meniscus–cartilage angle was measured on the coronal image demonstrating the central, best-defined section of the medial meniscal body and medial tibiofemoral compartment, avoiding more anterior or posterior sections where partial-volume or meniscal deformation effects are more prominent. A line tangent to the superior surface of the medial meniscus and a second line tangent to the adjacent medial tibial articular cartilage surface at the same level were constructed, and the medial meniscus–cartilage angle was recorded as the angle between these two lines. This parameter was evaluated together with medial tibial slope and medial tibial depth to provide complementary information regarding medial compartment morphology.

### 2.6. Measurement Reliability and Bias Control

Several measures were incorporated to minimize potential sources of bias. Identical eligibility criteria and standardized morphometric definitions were applied across the study groups. Anatomical landmarks and measurement procedures were defined before the comparative and machine-learning analyses, and the same measurement framework was used for ACL-injured and control knees. Independent image assessment by two experienced orthopedic surgeons was used to reduce dependence on a single observer. To formally evaluate the reproducibility of the morphometric measurements, interobserver agreement between the two observers, and intraobserver agreement for each observer, were assessed for all 18 parameters using the intraclass correlation coefficient (ICC), calculated with a two-way random-effects, absolute-agreement, single-measurement model.

Potential analytical bias was addressed through strict separation of model training and validation procedures. For classifiers sensitive to feature scale (support vector machine (SVM) and k-nearest neighbors (kNN)), predictors were standardized (zero mean, unit variance) using the mean and standard deviation estimated exclusively from the training fold of each cross-validation iteration; the same transformation was then applied to the corresponding validation fold. For least absolute shrinkage and selection operator (LASSO)-based feature selection, predictor standardization was performed internally by the glmnet implementation (default behavior of cv.glmnet), computed exclusively within the training fold, with resulting coefficients returned on the original predictor scale. Logistic regression, random forest (RF), naive Bayes, and XGBoost are not scale-dependent and were applied to unscaled predictors. All data-dependent preprocessing and feature-selection procedures were performed within the training data of each cross-validation iteration rather than on the complete dataset, and no scaling parameters were estimated from, or applied to, data outside the corresponding training fold at any stage.

### 2.7. Statistical Analysis

All statistical and machine learning analyses were performed in R software version 4.4.2 (R Foundation for Statistical Computing, Vienna, Austria). Eighteen morphological knee parameters were compared between the ACL and control groups using the Mann–Whitney U test or Welch’s *t*-test, selected according to Shapiro–Wilk normality testing, with Benjamini–Hochberg false discovery rate (FDR) correction applied across the 18 comparisons. To identify morphometric features discriminative of ACL injury status, six feature-selection (FS) methods (correlation filter, ReliefF [15], LASSO [16], random forest importance, recursive feature elimination [RFE] [17], and Boruta [18]) were combined with six classifiers (logistic regression, random forest, support vector machine, k-nearest neighbors, naive Bayes, and XGBoost), yielding 36 FS-classifier combinations. Each combination was evaluated using repeated 10-fold cross-validation (10 repeats, 100 folds total) [19], with feature selection performed independently within each training fold to prevent data leakage. All classifiers used fixed, pre-specified hyperparameters rather than cross-validated tuning: Random Forest (ntree = 500, default mtry), XGBoost (max_depth = 3, learning rate = 0.1, subsample = 0.8, colsample_bytree = 0.8, nrounds = 100), Support Vector Machine (radial basis kernel, cost = 1, gamma = 1/p, where p is the number of selected predictors), and k-Nearest Neighbors (k = 5); Logistic Regression and Naive Bayes used their standard default settings. No hyperparameter was selected using validation-fold or full-dataset information—all values were fixed prior to model development. Feature selection and model fitting were performed exclusively on the training partition of each outer fold; the held-out fold was used only for prediction and was never involved in feature selection, hyperparameter values, or any preprocessing statistic (e.g., scaling parameters were estimated from the training fold only and applied to the held-out fold). Because hyperparameters were fixed a priori rather than tuned across folds (see above), no tuning-related information from the outer evaluation data could have influenced model configuration. The only internal cross-validation used during model development was LASSO’s λ selection (glmnet::cv.glmnet, 5-fold internal cross-validation), which was itself confined to the training partition of that outer fold. This workflow is therefore equivalent to a nested cross-validation design, in which the inner loop never accesses outer-fold test data. Three of the six feature-selection methods used a fixed selection size of k = 9 by design: a Spearman correlation filter (top 9 predictors by absolute correlation with the outcome), ReliefF (k = 10 nearest neighbors used for weight estimation; top 9 features retained by weight), and Random Forest Gini importance (ntree = 500; top 9 features by mean decrease in Gini index). The remaining three methods determined the number of selected features adaptively: LASSO retained predictors with non-zero coefficients at λ.1se (glmnet::cv.glmnet, alpha = 1, 5-fold internal cross-validation within the training fold only; if fewer than two predictors were retained, the method defaulted to the top 3 by correlation); Recursive Feature Elimination (caret::rfe with a random-forest base learner) selected the subset size from candidate sizes {3, 6, 9, 12, 15, 18} via internal 3-fold cross-validation; and Boruta (maxRuns = 100) used its confirmed/rejected permutation-based statistical test with no fixed target size, defaulting to the top 3 by correlation only if fewer than two features were confirmed. Discrimination performance was assessed via the area under the receiver operating characteristic curve (AUC) and, at a fixed 0.5 probability threshold, accuracy, sensitivity, specificity, positive predictive value (PPV), negative predictive value (NPV), F1-score, and Matthews correlation coefficient (MCC). Accuracy, sensitivity, specificity, PPV, NPV, F1, and MCC were calculated using a fixed threshold of 0.5, chosen a priori as a clinically neutral default requiring no data-driven optimization. A secondary analysis additionally reports sensitivity and specificity at a Youden-optimal threshold, derived from the pooled out-of-fold predictions across all 100 outer folds; this threshold is presented as a secondary, exploratory operating point rather than the primary decision rule. We note that because this threshold was both derived from and evaluated on the same pooled out-of-fold predictions, the corresponding sensitivity and specificity estimates may carry a modest degree of optimism. A Friedman test followed by Nemenyi post hoc pairwise comparisons on the fold-level AUC values was used to identify the subset of methods not statistically significantly different from the best-performing combination (“top-tier” methods). For the top-tier methods, calibration was assessed on pooled out-of-fold predicted probabilities using the Brier score and logistic recalibration (calibration slope and intercept) [20], and the Youden-optimal classification threshold was derived, with sensitivity and specificity recomputed at this threshold. Feature selection stability across the 100 folds was quantified for each of the six FS methods using the Nogueira stability index [21] and the average pairwise Jaccard similarity between selected feature subsets. All 18 morphometric parameters were fully available for all 462 participants (233 ACL-injured, 229 controls), with no missing values for any parameter; therefore, no imputation procedure was required. Given the retrospective design, sample size was determined by the number of eligible patients meeting the inclusion criteria during the study period rather than by a priori power calculation. The interval reported alongside each AUC value represents the 2.5th–97.5th percentile range of the 100 fold-level AUC estimates from repeated cross-validation, describing observed variability across resampling iterations. Because the underlying folds are not statistically independent, this range should not be interpreted as a conventional confidence interval for the population mean AUC. As a complementary, formally valid estimate of the precision of the mean AUC, we additionally computed a 95% confidence interval using the Nadeau–Bengio corrected variance estimator for repeated k-fold cross-validation [22,23], which explicitly accounts for the dependence introduced by overlapping training/validation partitions across folds. To provide a principled basis for model development, the events-per-variable (EPV) ratio was calculated for the 18 candidate morphometric parameters: with 233 ACL-injured cases, the EPV was 12.9, exceeding the widely recommended minimum threshold of 10 for multivariable model development [24]. To evaluate whether the observed imbalance in knee laterality between groups could materially affect the findings, two sensitivity analyses were performed. First, each of the 18 morphometric parameters was compared between left- and right-sided knees using the same testing procedure described above, with Benjamini–Hochberg correction. Second, a multivariable logistic regression model for ACL injury status was fitted with and without laterality as an additional covariate, and model discrimination (AUC) and the statistical significance of the laterality term were compared between the two models. A parallel analysis was performed for sex: the 18 morphometric parameters were compared between male and female patients using the same procedure, and a multivariable logistic regression model for ACL injury status was fitted with and without sex as an additional covariate. The following package versions were used for the principal analytical steps: caret 7.0-1 (repeated cross-validation via createMultiFolds), glmnet 4.1-9 (LASSO), randomForest 4.7-1.2, e1071 1.7-16 (SVM), Boruta 8.0.0, xgboost 1.7.11.1, and pROC 1.19.0.1 (AUC and calibration metrics). A fixed random seed (set.seed(2026)) was used prior to all resampling and model-fitting procedures to ensure that the cross-validation fold assignments and reported results are exactly reproducible when the analysis is rerun with the same seed.

## 3. Results

**Figure 1:**
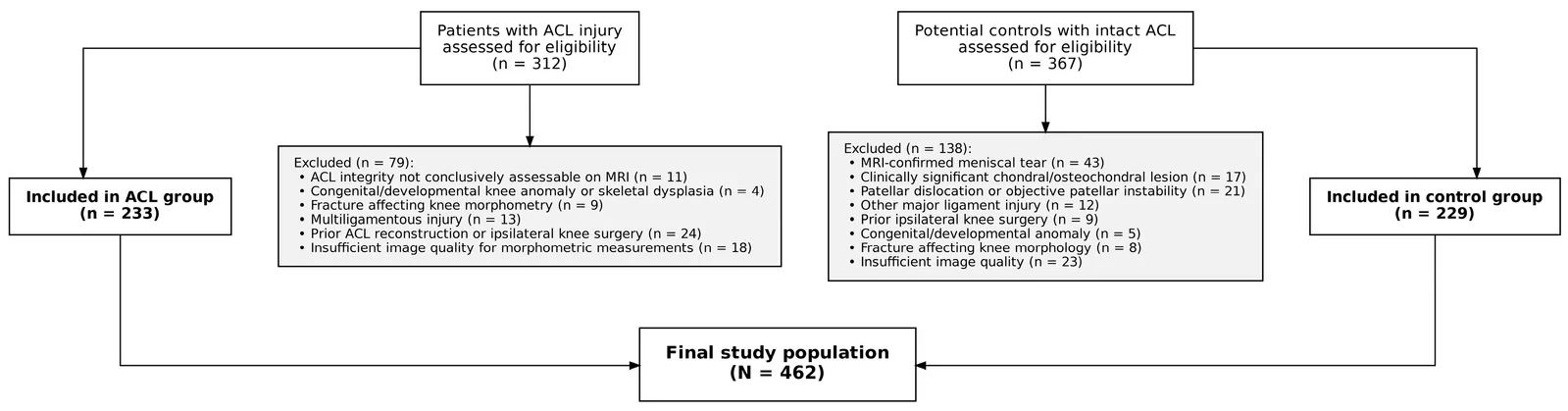
Flow diagram of patient selection for the anterior cruciate ligament (ACL)-injury and control groups.

Of 679 patients initially assessed for eligibility (312 with ACL injury and 367 potential controls), 217 were excluded according to predefined criteria (Figure 1), yielding a final cohort of 462 pediatric patients: 233 with anterior cruciate ligament (ACL) injury and 229 uninjured controls (Table 1). The two groups were well balanced for age (mean 15.39 vs. 15.20 years; Welch’s *t*-test *p* = 0.204, Mann–Whitney U *p* = 0.153) and sex distribution (85.0% vs. 86.9% male; *p* = 0.646). Side distribution differed significantly between groups (*p* = 0.026), with a higher proportion of left-sided involvement in the ACL group (45.1%) than in the control group (34.5%). Interobserver reliability was excellent for all 18 morphometric parameters (ICC 0.92–0.97; 95% CI, 0.905–0.975), as was intraobserver reliability for both observers (ICC 0.979–0.991 and 0.981–0.990, respectively; Supplementary Table S2).

Of the 18 morphological parameters, 13 differed significantly between the ACL and control groups after FDR correction (*p* < 0.05; Table 2). The largest effect sizes were observed for the axial intercondylar notch angle (Cohen’s d = 1.24), lateral femoral condyle ratio (rank-biserial r = −0.59), medial tibial depth (r = −0.39), coronal intercondylar notch angle (d = −0.39), and medial tibial slope (r = −0.38), indicating that notch geometry and tibial slope parameters were the most discriminative individual features. Five parameters did not reach significance after FDR correction: notch width, proximal tibial AP distance, notch/eminence ratio, notch height, and coronal lateral wall angle. The coronal lateral wall angle showed a nominally significant uncorrected *p*-value (*p* = 0.042), but this difference did not remain significant after FDR correction (p(FDR) = 0.054). Laterality was significantly associated with only 1 of the 18 morphometric parameters after FDR correction (lateral femoral condyle anteroposterior distance; p_FDR = 0.036), with a small absolute difference between sides (median 62.9 vs. 64.1 mm). In the multivariable logistic regression model, laterality was not significantly associated with ACL injury status after adjustment for all 18 morphometric parameters (OR 1.02, 95% CI 0.49–2.13, *p* = 0.965), and its inclusion did not materially change model discrimination (AUC 0.9656 vs. 0.9657 with laterality included; likelihood ratio test *p* = 0.965). These findings indicate that the laterality imbalance between groups is unlikely to have materially influenced the morphometric comparisons or classification results. Sex was significantly associated with 7 of the 18 morphometric parameters after FDR correction, predominantly size-related measurements (e.g., intercondylar notch width, lateral femoral condyle anteroposterior distance, proximal tibial anteroposterior distance), consistent with known sex-related differences in knee dimensions. However, sex was not significantly associated with ACL injury status in the multivariable logistic regression model after adjustment for all 18 morphometric parameters (OR 1.94, 95% CI 0.62–6.19, *p* = 0.258), and its inclusion did not materially change model discrimination (AUC 0.9656 vs. 0.9664 with sex included; likelihood ratio test *p* = 0.255). A fully powered sex-stratified sensitivity analysis restricted to female patients was not feasible given the limited female sample size (*n* = 65; events-per-variable = 1.67 for an 18-parameter model, well below the recommended threshold of 10). To evaluate whether the observed morphometric differences were confounded by age or sex, all 18 parameters were additionally compared using linear regression models adjusting for age and sex (ANCOVA), together with a rank-based sensitivity analysis for parameters violating the normality assumption. All 13 parameters that remained significant after FDR correction in the unadjusted comparison also remained significant after age- and sex-adjustment in both the ANCOVA and the rank-based sensitivity analysis (Supplementary Table S3), indicating that the observed group differences are not attributable to the modest age or sex distributional differences between the ACL and control groups.

**Figure 2:**
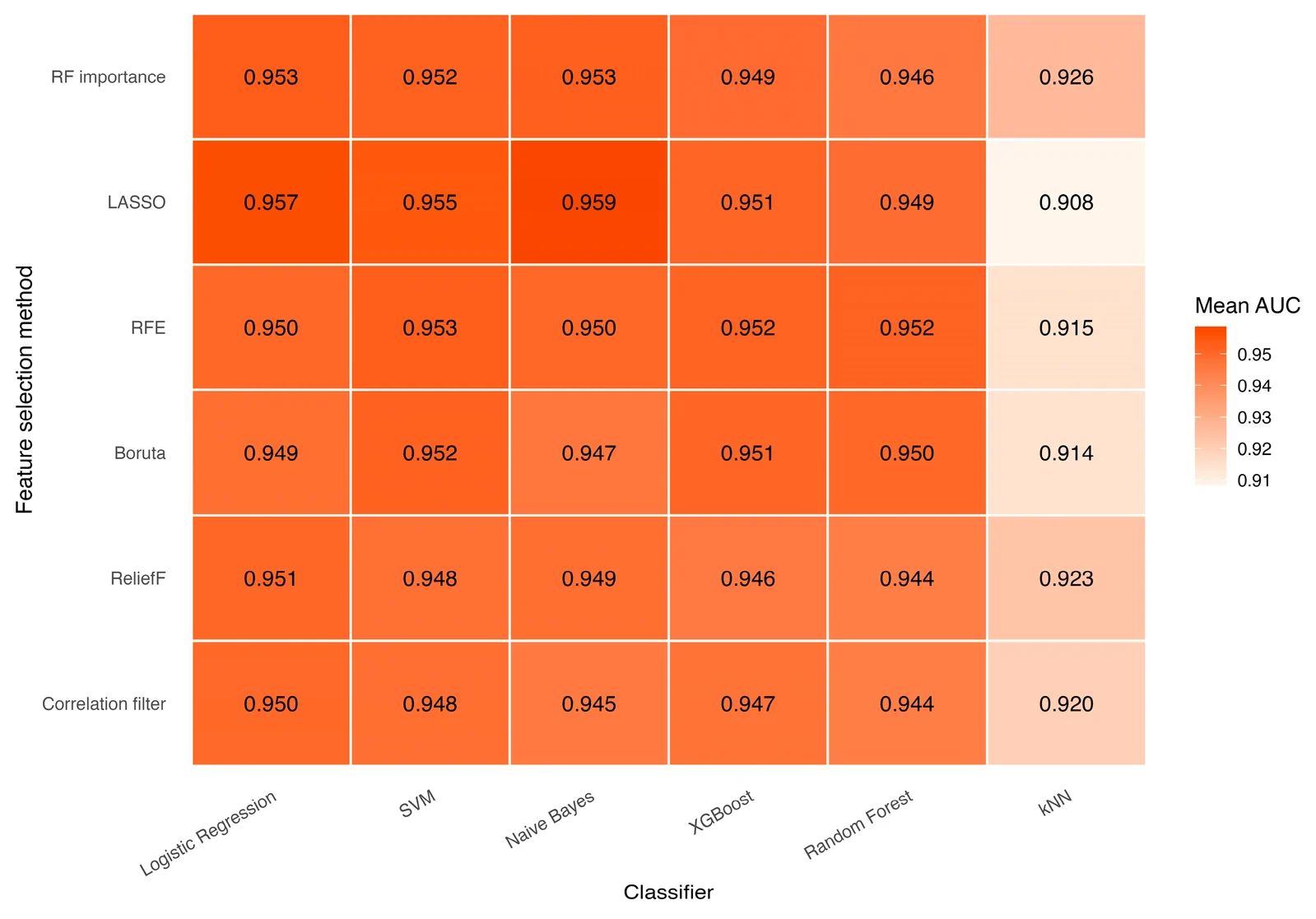
Mean AUC heatmap across feature selection methods and classifiers.

Across the 36 feature selection × classifier combinations, mean AUC ranged from 0.908 to 0.959, accuracy from 0.817 to 0.897, sensitivity from 0.726 to 0.899, and MCC from 0.649 to 0.797 (Table 3, Supplementary Table S4; Figure 2).

**Figure 3:**
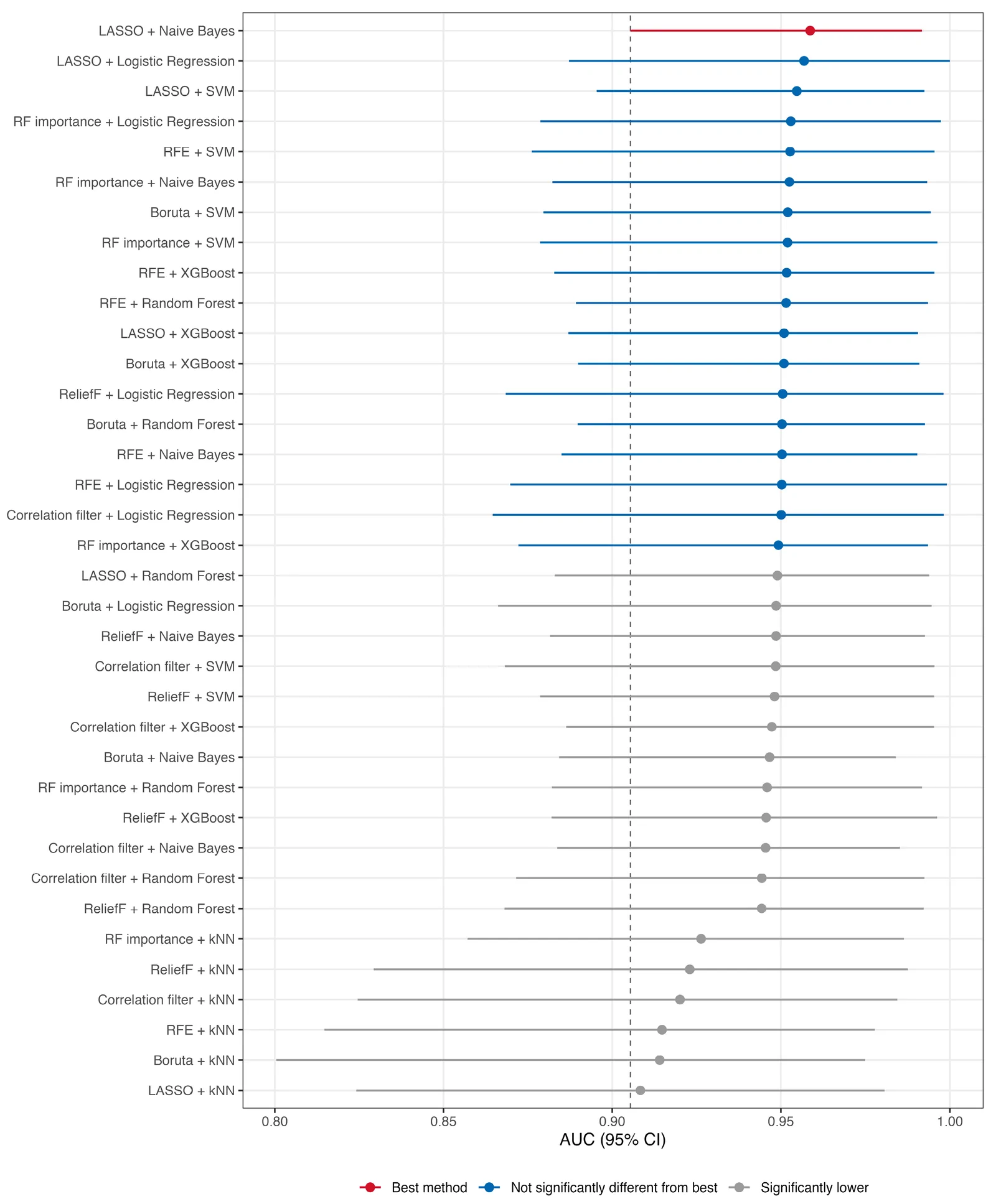
Forest plot of AUC by feature selection–classifier combination. The dashed vertical line indicates the lower bound (AUC = 0.905) of the 95% cross-validation range for the best-performing model.

The best-performing combination was LASSO + Naive Bayes (AUC 0.959; 95% range across 100 CV iterations, 0.905–0.992; Nadeau–Bengio corrected 95% CI, 0.942–0.976 [22,23]). A Friedman test indicated a significant overall difference among the 36 methods (χ^2^ = 894.2, df = 35, *p* < 0.001). Nemenyi post hoc testing identified 18 of 36 combinations as not significantly different from the best method (*p* > 0.05), forming a statistically indistinguishable top tier (Figure 3); the remaining 18 combinations, most of which used kNN or the correlation filter/ReliefF feature selectors, performed significantly worse. Among the top-tier methods, RFE (5 combinations), and LASSO and RF importance (4 each) were the most frequently represented feature selection methods, while logistic regression (5 combinations) was the most frequently represented classifier, followed by SVM and XGBoost (4 each).

**Figure 4:**
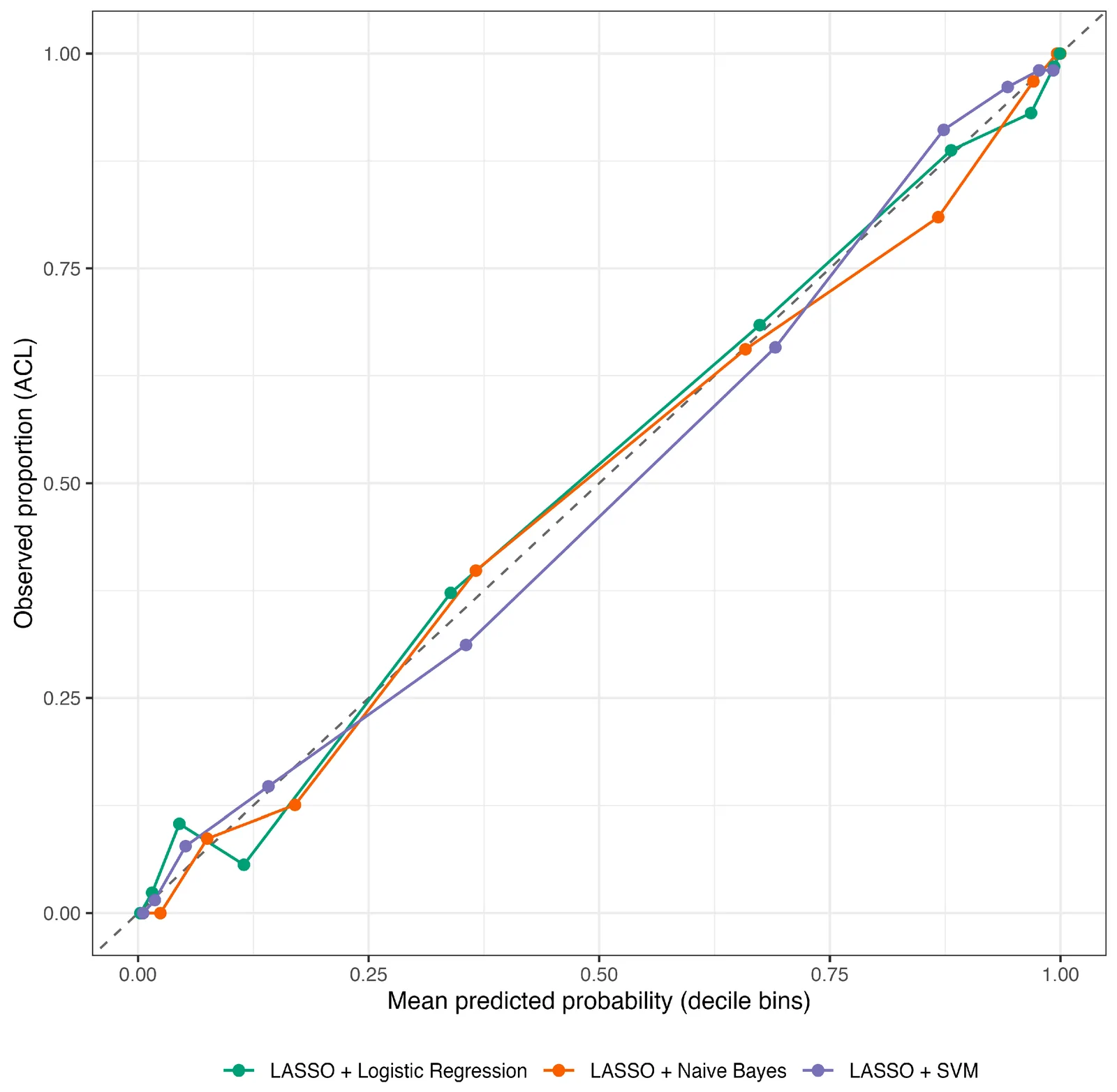
Calibration plot of the top-performing models. The dashed diagonal line represents perfect calibration (observed proportion = predicted probability).

Among the 18 top-tier methods, calibration—assessed under this case–control cohort’s approximately 1:1 ACL:control sampling ratio rather than the true population prevalence of pediatric ACL injury—was generally acceptable for the Logistic Regression-, SVM-, and Naive Bayes-based combinations, with Brier scores ranging from 0.080 to 0.100 and calibration slopes mostly close to 1 (Table 4, Figure 4). In contrast, Random Forest-based combinations showed calibration slopes near 2.0 (e.g., RFE + Random Forest: 1.99; Boruta + Random Forest: 1.98), indicating a substantial departure from ideal calibration despite comparable discrimination. LASSO + Logistic Regression achieved the lowest Brier score among the top-tier methods (Brier = 0.080, slope = 0.91, intercept = 0.01), while LASSO + SVM showed comparably favorable calibration, with a slope and intercept closer to the ideal values of 1 and 0 (Brier = 0.080, slope = 1.00, intercept = −0.01). In contrast, RFE combined with Random Forest or Naive Bayes showed substantial calibration slope departures from 1 (≈1.99 and 0.79, respectively), suggesting less reliable probability estimates despite comparable discrimination. At the Youden-optimal threshold, sensitivity and specificity for the top-tier methods ranged from approximately 0.84–0.92 and 0.87–0.91, respectively.

**Figure 5:**
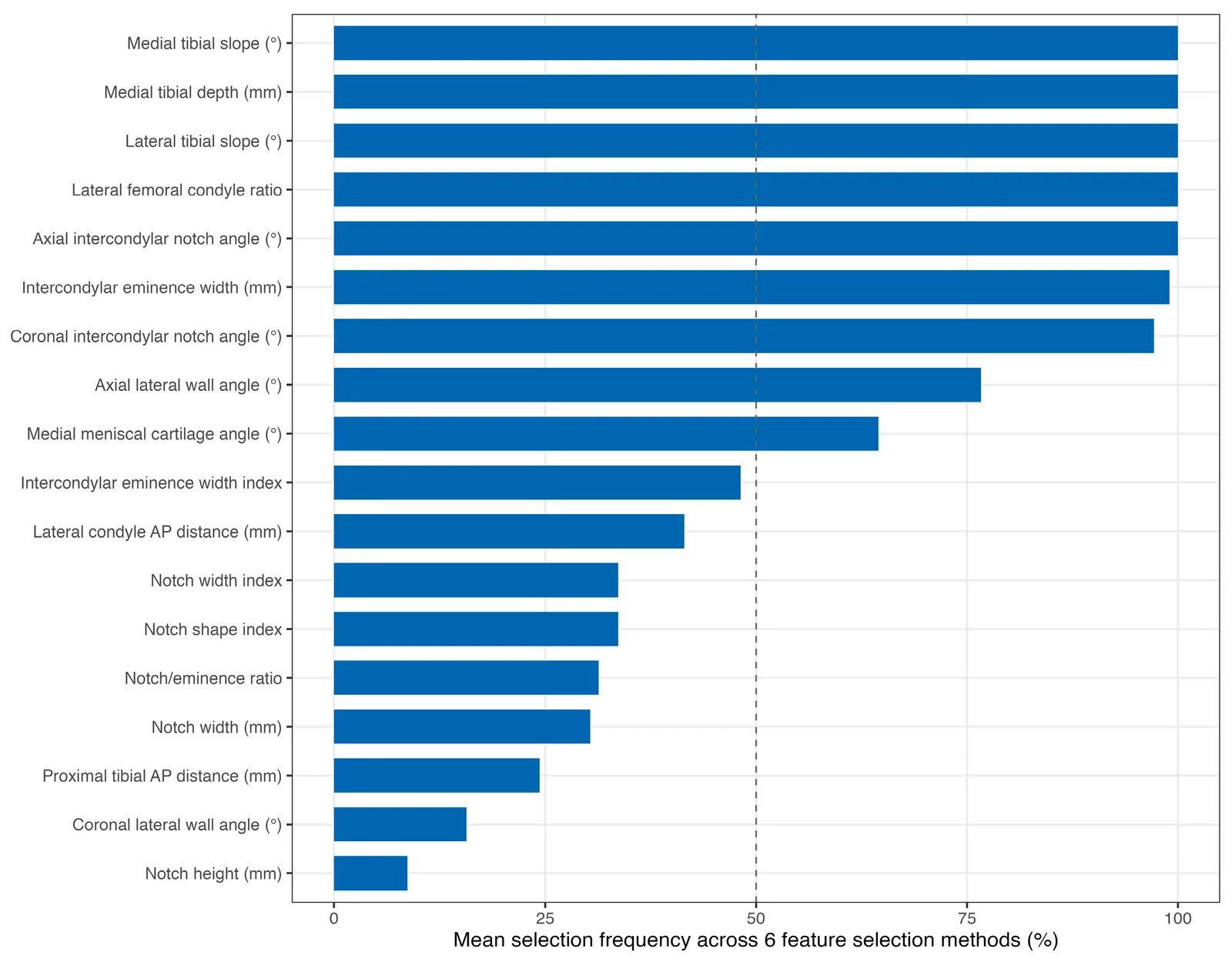
Feature selection consensus across morphological parameters. The dashed vertical line indicates a 50% mean selection frequency across the six feature-selection methods.

Feature selection stability varied considerably across the six FS methods (Table 5, Figure 5). LASSO was the most stable (Nogueira stability index = 0.985, mean Jaccard similarity = 0.986), consistently selecting a core of 8 parameters across all 100 folds, with a narrow range of 8–10 features selected per fold. Correlation filter, ReliefF, and RF importance showed intermediate-to-high stability (Nogueira index 0.85–0.91), each selecting a fixed 9 features per fold with 6–7 core parameters. Boruta showed moderate stability (Nogueira index = 0.594), selecting a substantially larger and more variable feature set (mean 14.6, range 12–17) with 11 core parameters. RFE was the least stable (Nogueira index = 0.171), reflecting substantial fold-to-fold variability in both the number (6–18) and identity of selected features. Computationally, correlation filter and LASSO were the fastest methods (mean runtime 0.001 and 0.029 s per fold, respectively), while Boruta was markedly slower (8.656 s per fold), reflecting its iterative random forest-based selection procedure. Despite this variability in stability and runtime, five parameters—axial intercondylar notch angle, lateral femoral condyle ratio, lateral tibial slope, medial tibial depth, and medial tibial slope—were selected in 100% of folds by all six feature selection methods, indicating strong consensus on their discriminative relevance regardless of the selection approach used.

## 4. Discussion

The principal finding of this study was that pediatric ACL injury was associated with a distinct, multidimensional pattern of knee morphology on MRI. In total, 13 of the 18 evaluated morphometric parameters differed significantly between ACL-injured and control knees after correction for multiple comparisons, indicating that the anatomical differences between groups were not confined to a single structure or imaging plane. In parallel, machine-learning models integrating these morphometric features demonstrated excellent discrimination, with mean AUC values ranging from 0.908 to 0.959. Although LASSO combined with naive Bayes achieved the numerically highest discrimination, 18 of the 36 feature-selection/classifier combinations formed a statistically indistinguishable top tier. Of particular relevance, LASSO demonstrated remarkably high feature-selection stability and consistently identified a core set of anatomical characteristics across repeated resampling. These findings support the concept that pediatric ACL injury is associated not simply with an isolated anatomical abnormality but with a reproducible morphometric pattern involving both tibial and femoral geometry.

The differences observed in tibial morphology are consistent with previous evidence implicating tibial plateau geometry in ACL injury. Increased posterior tibial slope may favor anterior translation of the tibia relative to the femur and thereby increase loading of the ACL during dynamic activity. Previous MRI-based studies have associated both medial tibial depth and medial and lateral tibial slopes with ACL injury [8], while pediatric case–control data have specifically demonstrated an association between increased lateral tibial slope and ACL injury [9]. The present findings extend these observations by demonstrating simultaneous differences in medial tibial slope, lateral tibial slope, and medial tibial depth within the same pediatric cohort. This pattern suggests that the geometry of the tibial plateau may be better understood as an integrated anatomical construct rather than as a series of independent measurements.

Intercondylar notch morphology represents another important component of this anatomical profile. Previous pediatric MRI studies have demonstrated an association between intercondylar notch dimensions and ACL injury in skeletally immature patients [10]. More recent pediatric evidence has reinforced the relevance of both tibial slope and notch morphology. Shin et al. reported that a high articular lateral tibial slope was associated with pediatric ACL tears and that patients with ACL tears had a narrower intercondylar notch than comparison groups [25]. Edwards et al. similarly showed that lateral posterior tibial slope and its relationship with lateral meniscal geometry were associated with pediatric ACL injury, illustrating the importance of considering multiple interacting anatomical characteristics rather than a single measurement [26].

The significance of intercondylar notch dimensions in pediatric ACL injury has also been demonstrated across different study designs. Yellin et al., in a multicenter pediatric study, reported significantly smaller notch width indices among patients with unilateral and bilateral ACL rupture compared with controls [27]. Importantly, pediatric notch morphology itself changes during growth. Lima et al. demonstrated that intercondylar notch width increases predominantly during the earlier years of childhood, subsequently stabilizes during early adolescence, and shows age- and sex-related developmental patterns [28]. These observations are particularly relevant to the present study because they emphasize that osseous morphology in pediatric populations should not be interpreted as a static adult-like characteristic. The significant differences identified in notch width index, notch shape index, and angular measurements in our cohort may therefore reflect a combination of individual anatomical susceptibility and developmental variation.

Recent pediatric MRI studies have further suggested that conventional notch width alone may not capture the full geometric characteristics associated with ACL injury. Manhard et al. demonstrated differences in several osseous and cartilaginous notch-related dimensions between pediatric patients with and without ACL injury and proposed that vertical characteristics of the notch may provide additional information beyond conventional NWI [29]. Similarly, previous work evaluating notch configuration has suggested that angular characteristics may complement simple linear width measurements when assessing anatomical associations with ACL injury [30]. This concept is consistent with our findings, in which both coronal and axial intercondylar notch angles contributed to the morphometric differences between ACL-injured and control knees. Such findings support a more comprehensive representation of intercondylar notch geometry incorporating width, height, shape, and orientation rather than reliance on a single index.

Femoral condylar morphology also emerged as an important component of the anatomical differences observed in the present cohort. The LFCR demonstrated one of the most pronounced differences between ACL-injured and control knees. MRI-based evidence has shown that an increased LFCR is associated with noncontact ACL injury and that combining lateral femoral condylar geometry with other osseous characteristics can improve discrimination between injured and control knees. In the study by He and Li, LFCR demonstrated an AUC of 0.81 individually, which increased when combined with an additional anatomical characteristic, supporting the concept that combined morphology may be more informative than individual measurements [31]. More broadly, Barnum et al. demonstrated that combinations of geometric features of the knee were associated with noncontact ACL injury, further supporting the view that ACL susceptibility reflects a multidimensional anatomical environment [32].

This multidimensional nature provides a strong rationale for the use of machine-learning methods in the present study. Conventional univariable comparisons are useful for identifying individual group differences but may inadequately characterize relationships among correlated anatomical variables, nonlinear effects, and higher-order interactions. Machine learning offers a framework in which multiple anatomical features can be evaluated simultaneously while different approaches to feature selection and classification can be systematically compared. Recent work has begun to apply these approaches to ACL injury assessment, and current evidence suggests that machine-learning models can integrate heterogeneous risk factors with promising discriminatory performance. A recent systematic review and meta-analysis of machine-learning applications in ACL injury prediction similarly highlighted the increasing use of such methods while emphasizing substantial variation in input variables, model development, and validation strategies across studies [33].

The present study extends this literature by integrating a relatively broad panel of 18 femoral and tibial MRI-derived morphometric features in a specifically pediatric cohort, systematically comparing 36 feature-selection/classifier combinations within a common repeated cross-validation framework, and additionally evaluating the stability of feature selection across resampling iterations. Recent pediatric imaging studies have also demonstrated that ACL-injured children may exhibit combinations of differences in tibial slope, notch width index, medial tibial depth, and femoral morphology compared with normally developing controls [34]. Our findings extend this concept by showing that these multidimensional anatomical differences contain sufficient information to distinguish ACL-injured from control knees with consistently high discrimination across several fundamentally different algorithms. The fact that numerous classifier combinations achieved similar AUC values suggests that the observed discrimination was not dependent on a single modeling architecture and supports internal consistency of the morphometric signal within the present cohort. However, this should not be interpreted as evidence of external robustness, which requires validation in independent populations.

An additional clinically and methodologically relevant observation was that the model with the numerically highest AUC was not necessarily superior across all aspects of model performance. LASSO combined with naive Bayes achieved the highest mean AUC, whereas LASSO combined with logistic regression demonstrated excellent discrimination together with particularly favorable calibration. This distinction is important because discrimination describes a model’s ability to separate groups, whereas calibration reflects the agreement between predicted probabilities and observed outcomes. Furthermore, feature-selection stability differed substantially among methods. LASSO achieved a Nogueira stability index of 0.985 and a mean Jaccard similarity of 0.986, consistently retaining a core set of features across repeated resampling, whereas RFE exhibited considerably greater variability. These findings suggest that marginal differences in AUC should not be considered the sole criterion for identifying a useful analytical strategy. In studies seeking to characterize anatomical phenotypes, reproducibility of feature selection may be equally important because unstable feature sets can substantially limit biological interpretation and external reproducibility.

Skeletal maturity represents an additional consideration when interpreting these findings. Although the ACL-injured and control groups were comparable in chronological age, children of similar ages may differ in their degree of skeletal maturation. Because knee morphology continues to change during growth, unmeasured differences in skeletal maturity may have contributed to variability in the morphometric parameters and may partly influence the anatomical differences observed between groups. Accordingly, the morphometric profile identified in the present study should not be interpreted as independent of skeletal maturation. Future studies incorporating formal measures of skeletal maturity may help distinguish injury-associated anatomical characteristics from morphology related to normal developmental variation.

The clinical interpretation of these results requires caution. The present classification framework should not be interpreted as providing an immediate diagnostic advantage for established ACL rupture, because ACL disruption can generally be directly identified on conventional MRI. Rather, the principal value of the analysis lies in characterizing a multidimensional morphometric phenotype associated with ACL injury status and in identifying anatomical features that consistently contribute to discrimination between injured and control knees. In this context, the machine-learning framework serves primarily as a tool for integrating correlated morphometric information rather than as a substitute for routine MRI diagnosis of ACL injury. The present models distinguish knees with established ACL injury from control knees on the basis of MRI-derived anatomy; they do not prospectively predict which currently uninjured child will subsequently sustain an ACL rupture. Therefore, the identified morphometric characteristics should be interpreted as anatomical features associated with ACL injury status, rather than deterministic or prospectively validated risk factors. Accordingly, the high discrimination observed in this case–control setting reflects the ability to distinguish established injury status and should not be interpreted as evidence that the models can estimate an individual patient’s future probability of ACL injury. Prospective longitudinal validation in initially uninjured pediatric cohorts would be required before any risk-prediction or screening application could be considered. Nevertheless, defining a reproducible morphometric phenotype may provide a foundation for future longitudinal studies. Any future evaluation of individualized ACL injury risk would require prospective longitudinal cohorts of initially uninjured participants, ideally with pre-injury imaging and integration of formal skeletal-maturity assessment, sport exposure, neuromuscular control, lower-limb alignment, and biomechanical variables, together with independent multicenter external validation.

The generalizability of the present models also requires consideration. Because the study was conducted at a single institution using a consistent MRI acquisition and measurement framework, the observed model performance may partly reflect characteristics specific to the study population and imaging environment. Variations across institutions in patient demographics, scanner characteristics, acquisition protocols, image quality, and image-plane selection may affect morphometric measurements and consequently influence model performance. Therefore, internal cross-validation, although useful for estimating performance within the present dataset, cannot establish transportability to external clinical settings. Independent multicenter validation across heterogeneous pediatric populations and MRI protocols will be essential to determine whether the identified morphometric profile and associated classification performance remain reproducible under different clinical and imaging conditions.

### Limitations

Several limitations should be considered when interpreting the present findings. The retrospective, single-center case–control design may have introduced selection bias and may limit the generalizability of the results to other institutions and pediatric populations. In addition, the control group consisted of clinically referred patients with intact ACLs rather than a population-based healthy cohort. Accordingly, the control population may not fully represent the underlying healthy pediatric population, and some degree of spectrum or referral bias cannot be excluded. Although model performance was evaluated using extensive repeated cross-validation, an independent external validation cohort was not available. The reported discrimination therefore reflects internal model performance and should not be assumed to translate directly to other clinical settings, imaging protocols, or patient populations. The case–control sampling strategy also does not reflect the population prevalence of ACL injury. Consequently, predicted probabilities and calibration estimates should not be interpreted as estimates of absolute ACL injury risk in the general pediatric population. Pediatric knee morphology changes throughout skeletal development, and chronological age does not fully capture skeletal maturity. Formal measures of skeletal maturation or pubertal stage were not incorporated into the present analysis. Therefore, residual confounding related to differences in skeletal development between individuals of similar chronological age cannot be excluded. Skeletal maturity indices (e.g., Tanner stage or bone age) were not recorded in this cohort and could not be incorporated into the age/sex-adjusted analysis; skeletal maturity may capture growth-related variation in knee morphometry beyond what chronological age alone reflects, and its absence remains a limitation of this and related pediatric morphometric studies. Furthermore, the machine-learning models were intentionally restricted to the 18 MRI-derived morphometric characteristics and did not include potentially relevant demographic, biomechanical, neuromuscular, or sport-related variables. Although this approach allowed the discriminatory information contained specifically within knee morphology to be evaluated, ACL injury is multifactorial, and future models incorporating these additional domains may provide a more comprehensive representation of injury susceptibility. Because the repeated cross-validation folds are not statistically independent—the same patients recur across training and validation partitions in different folds—the Friedman test’s exchangeability assumption is only approximately satisfied. Nominal *p*-values from the Friedman/Nemenyi procedure should therefore be interpreted as a descriptive ranking of relative model performance rather than as strictly valid inferential statistics derived from independent samples. This is a recognized limitation of applying classical rank-based tests to resampling-based model comparisons [35,36]. The morphometric variables were derived from two-dimensional MRI measurements and therefore remain dependent on image-plane selection and identification of anatomical landmarks. Despite the use of standardized measurement definitions and experienced observers, some measurement variability is unavoidable. These factors may affect the reproducibility of individual morphometric measurements across different clinical environments. The cohort was predominantly male (85.9%). Although sex was not significantly associated with ACL injury status after adjustment for the morphometric parameters, and its inclusion did not materially affect model discrimination, several individual parameters differed significantly by sex, consistent with known sex-related differences in knee anatomy. Given the limited number of female participants, a fully powered sex-stratified sensitivity analysis was not feasible, and the generalizability of the model to female patients could not be directly confirmed; this should be addressed in future studies with adequate female representation. Although clinical information, radiology reports, and group labels were withheld from observers and cases were assessed in a randomized order, complete blinding to ACL status was not achievable, as ACL disruption may be directly or indirectly visible on MRI, including on the sagittal sequences used for several measurements. This introduces the possibility of residual observer bias. However, the inter- and intraobserver reliability observed across all 18 parameters (interobserver ICC 0.92–0.97; intraobserver ICC 0.98–0.99) indicates that measurements remained highly reproducible, suggesting that any residual bias had limited practical impact on the morphometric measurements themselves. Finally, the cross-sectional case–control nature of the study precludes determination of temporal or causal relationships between the observed anatomical characteristics and ACL injury. The models were developed to discriminate knees with established ACL injury from controls and should not be interpreted as prospective models for predicting future ACL rupture. Prospective multicenter studies with external validation, formal assessment of skeletal maturity, and longitudinal follow-up are required to determine whether the identified morphometric profile has value in future individualized risk-stratification strategies.

## 5. Conclusions

Pediatric ACL injury was associated with a distinct multidimensional MRI-based morphometric profile involving both tibial and femoral anatomy. Multiple machine-learning approaches demonstrated high discrimination between ACL-injured and control knees, while LASSO-based feature selection showed particularly high reproducibility across repeated resampling. These findings suggest that the anatomical differences associated with pediatric ACL injury are distributed across a combination of morphometric characteristics rather than being attributable to a single measurement. The present models should be interpreted as classifiers of established ACL injury status rather than prospective predictors of future ACL rupture. Prospective, multicenter studies with external validation are needed to determine whether the identified morphometric profile is reproducible across different pediatric populations and whether its integration with skeletal maturity, demographic, biomechanical, and sport-related factors can contribute to future individualized ACL injury risk assessment.

## Figures and Tables

**Table 1:** Demographic characteristics of the ACL and control groups.

Characteristic	ACL Group (n = 233)	Control Group (n = 229)	*p*-Value
n	233	229	
Age, years—mean (SD)	15.39 (1.57)	15.20 (1.57)	*p* = 0.153 (Mann–Whitney U)
Age, years—median [min–max]	16 [8–18]	16 [9–17]	
Sex—female, *n* (%)	35 (15.0%)	30 (13.1%)	0.6456
Sex—male, *n* (%)	198 (85.0%)	199 (86.9%)	
Side—right, *n* (%)	128 (54.9%)	150 (65.5%)	0.0261
Side—left, *n* (%)	105 (45.1%)	79 (34.5%)	

Data are *n*, mean (SD), median [min–max], or *n* (%). Age was compared using Welch’s two-sample *t*-test (Mann–Whitney U shown as sensitivity check in the main text). Sex and side were compared using the chi-square test. ACL = anterior cruciate ligament injury group; SD = standard deviation. For age, normality was assessed using the Shapiro–Wilk test in both groups (control: *p* < 0.001; ACL: *p* < 0.001); given the non-normal distribution, the Mann–Whitney U test was used as the pre-specified primary test, consistent with the normality-gated test selection used in Table 2. The Welch *t*-test result (*p* = 0.204) is reported here as a concordant sensitivity analysis: *t*-test *p* = 0.204.

**Table 2:** Comparison of 18 morphological parameters between the ACL and control groups.

Parameter	ACL (*n* = 233)	Control (*n* = 229)	Test	*p*	p (FDR)	Effect Size
Notch width (mm)	16.00 [13.90–18.10]	16.40 [14.80–18.10]	Mann–Whitney U	0.071	0.085	rank-biserial r = 0.10
Notch width index	0.19 [0.17–0.22]	0.20 [0.18–0.22]	Mann–Whitney U	0.015	0.021	rank-biserial r = 0.13
Lateral tibial slope (°)	7.80 [4.30–11.60]	5.60 [3.00–8.00]	Mann–Whitney U	<0.001	<0.001	rank-biserial r = −0.29
Medial tibial slope (°)	8.50 [5.20–11.40]	5.60 [3.00–8.20]	Mann–Whitney U	<0.001	<0.001	rank-biserial r = −0.38
Medial meniscal cartilage angle (°)	27.10 [23.80–30.80]	25.50 [22.80–28.80]	Mann–Whitney U	<0.001	<0.001	rank-biserial r = −0.19
Medial tibial depth (mm)	2.90 [2.40–3.80]	2.40 [2.00–3.00]	Mann–Whitney U	<0.001	<0.001	rank-biserial r = −0.39
Lateral condyle AP distance (mm)	64.20 [61.00–66.90]	63.00 [59.70–65.60]	Mann–Whitney U	0.005	0.008	rank-biserial r = −0.15
Proximal tibial AP distance (mm)	37.60 [32.80–40.50]	37.40 [34.00–39.90]	Mann–Whitney U	0.933	0.933	rank-biserial r = −0.00
Intercondylar eminence width (mm)	10.70 [9.40–12.00]	11.40 [10.40–12.60]	Mann–Whitney U	<0.001	<0.001	rank-biserial r = 0.24
Intercondylar eminence width index	0.14 [0.12–0.16]	0.15 [0.14–0.16]	Mann–Whitney U	<0.001	<0.001	rank-biserial r = 0.22
Notch/eminence ratio	1.46 [1.27–1.65]	1.40 [1.28–1.57]	Mann–Whitney U	0.088	0.099	rank-biserial r = −0.09
Notch shape index	0.72 [0.64–0.78]	0.75 [0.67–0.83]	Mann–Whitney U	0.002	0.003	rank-biserial r = 0.17
Notch height (mm)	21.90 [19.80–24.00]	21.40 [19.80–23.80]	Mann–Whitney U	0.573	0.607	rank-biserial r = −0.03
Lateral femoral condyle ratio	0.64 [0.62–0.67]	0.60 [0.57–0.63]	Mann–Whitney U	<0.001	<0.001	rank-biserial r = −0.59
Coronal intercondylar notch angle (°)	54.78 ± 10.74	58.84 ± 10.33	*t*-test	<0.001	<0.001	Cohen d = −0.39
Axial intercondylar notch angle (°)	61.30 ± 13.27	47.55 ± 8.35	*t*-test	<0.001	<0.001	Cohen d = 1.24
Axial lateral wall angle (°)	22.90 [20.40–26.90]	21.60 [18.60–24.90]	Mann–Whitney U	<0.001	<0.001	rank-biserial r = −0.20
Coronal lateral wall angle (°)	25.94 ± 5.18	26.92 ± 5.12	*t*-test	0.042	0.054	Cohen d = −0.19

Data are median [Q1–Q3] for non-normally distributed variables (Shapiro–Wilk *p* < 0.05 in either group) or mean ± SD for normally distributed variables. p(FDR) = Benjamini–Hochberg false discovery rate-corrected *p*-value across 18 comparisons. Effect size: Cohen’s d for *t*-test comparisons; rank-biserial r for Mann–Whitney U comparisons. Rank-biserial r is signed such that a positive value indicates lower values in the ACL group relative to controls, and a negative value indicates higher values in the ACL group; for example, the negative r values for lateral and medial tibial slope reflect steeper (higher) slopes in the ACL group compared with controls.

**Table 3:** Discrimination performance of all 36 feature selection × classifier combinations, ranked by mean AUC.

Rank	Feature Selection	Classifier	AUC (95% Range Across 100 CV Iterations)	Accuracy	Sensitivity	Specificity	PPV	NPV	F1	MCC	Top-Tier
1	LASSO	Naive Bayes	0.959 (0.905–0.992)	0.883	0.887	0.879	0.885	0.887	0.885	0.769	Yes
2	LASSO	Logistic Regression	0.957 (0.887–1.000)	0.893	0.891	0.895	0.899	0.894	0.893	0.789	Yes
3	LASSO	SVM	0.955 (0.895–0.992)	0.892	0.898	0.886	0.891	0.898	0.893	0.787	Yes
4	RF importance	Logistic Regression	0.953 (0.879–0.997)	0.892	0.887	0.896	0.900	0.890	0.892	0.787	Yes
5	RFE	SVM	0.953 (0.876–0.995)	0.892	0.899	0.885	0.891	0.899	0.893	0.787	Yes
6	RF importance	Naive Bayes	0.953 (0.882–0.993)	0.872	0.873	0.872	0.878	0.873	0.873	0.748	Yes
7	Boruta	SVM	0.952 (0.880–0.994)	0.895	0.899	0.890	0.896	0.899	0.896	0.792	Yes
8	RF importance	SVM	0.952 (0.879–0.996)	0.890	0.893	0.887	0.892	0.894	0.891	0.783	Yes
9	RFE	XGBoost	0.952 (0.883–0.995)	0.878	0.875	0.882	0.885	0.877	0.878	0.759	Yes
10	RFE	Random Forest	0.952 (0.889–0.994)	0.879	0.890	0.868	0.877	0.888	0.882	0.762	Yes
11	LASSO	XGBoost	0.951 (0.887–0.991)	0.874	0.876	0.872	0.878	0.877	0.875	0.752	Yes
12	Boruta	XGBoost	0.951 (0.890–0.991)	0.877	0.873	0.880	0.884	0.876	0.877	0.756	Yes
13	ReliefF	Logistic Regression	0.951 (0.868–0.998)	0.888	0.884	0.893	0.897	0.887	0.888	0.780	Yes
14	Boruta	Random Forest	0.950 (0.890–0.993)	0.876	0.885	0.867	0.875	0.885	0.878	0.756	Yes
15	RFE	Naive Bayes	0.950 (0.885–0.990)	0.869	0.889	0.850	0.862	0.886	0.873	0.743	Yes
16	RFE	Logistic Regression	0.950 (0.870–0.999)	0.897	0.898	0.895	0.900	0.900	0.897	0.797	Yes
17	Correlation filter	Logistic Regression	0.950 (0.865–0.998)	0.890	0.885	0.894	0.897	0.888	0.890	0.782	Yes
18	RF importance	XGBoost	0.949 (0.872–0.994)	0.867	0.864	0.870	0.875	0.867	0.868	0.738	Yes
19	LASSO	Random Forest	0.949 (0.883–0.994)	0.869	0.877	0.862	0.869	0.876	0.871	0.742	No
20	Boruta	Logistic Regression	0.949 (0.866–0.995)	0.894	0.893	0.895	0.899	0.895	0.894	0.791	No
21	ReliefF	Naive Bayes	0.949 (0.882–0.993)	0.865	0.871	0.858	0.866	0.871	0.866	0.733	No
22	Correlation filter	SVM	0.948 (0.868–0.995)	0.887	0.892	0.881	0.888	0.892	0.888	0.776	No
23	ReliefF	SVM	0.948 (0.879–0.995)	0.891	0.898	0.883	0.890	0.899	0.892	0.785	No
24	Correlation filter	XGBoost	0.947 (0.886–0.995)	0.864	0.858	0.870	0.874	0.862	0.864	0.732	No
25	Boruta	Naive Bayes	0.947 (0.884–0.984)	0.858	0.877	0.838	0.850	0.874	0.861	0.719	No
26	RF importance	Random Forest	0.946 (0.882–0.992)	0.869	0.872	0.866	0.873	0.873	0.870	0.742	No
27	ReliefF	XGBoost	0.946 (0.882–0.996)	0.866	0.857	0.874	0.878	0.861	0.865	0.735	No
28	Correlation filter	Naive Bayes	0.945 (0.884–0.985)	0.864	0.882	0.845	0.857	0.879	0.867	0.732	No
29	Correlation filter	Random Forest	0.944 (0.872–0.992)	0.866	0.875	0.856	0.864	0.874	0.868	0.735	No
30	ReliefF	Random Forest	0.944 (0.868–0.992)	0.871	0.879	0.863	0.871	0.879	0.873	0.746	No
31	RF importance	kNN	0.926 (0.857–0.986)	0.855	0.813	0.899	0.893	0.830	0.848	0.717	No
32	ReliefF	kNN	0.923 (0.829–0.988)	0.856	0.810	0.902	0.897	0.828	0.848	0.718	No
33	Correlation filter	kNN	0.920 (0.825–0.984)	0.863	0.830	0.896	0.893	0.842	0.858	0.731	No
34	RFE	kNN	0.915 (0.815–0.978)	0.817	0.726	0.909	0.894	0.770	0.797	0.649	No
35	Boruta	kNN	0.914 (0.800–0.975)	0.823	0.738	0.910	0.895	0.778	0.806	0.660	No
36	LASSO	kNN	0.908 (0.824–0.981)	0.858	0.810	0.906	0.900	0.827	0.851	0.722	No

Results from repeated 10-fold cross-validation (10 repeats, 100 folds total); feature selection was performed independently within each training fold to prevent data leakage. All metrics except AUC were computed at a fixed probability threshold of 0.5 and are reported as the mean across the 100 folds. AUC = area under the receiver operating characteristic curve, reported as mean (95% range across 100 CV iterations); a complementary Nadeau–Bengio corrected 95% CI for the top-ranked combination is reported in the Results section. PPV = positive predictive value; NPV = negative predictive value; MCC = Matthews correlation coefficient. Top-tier = not statistically significantly different from the best-ranked method (Nemenyi post hoc *p* > 0.05, following a significant Friedman test: χ^2^ = 894.2, df = 35, *p* < 0.001; *n* = 18 of 36 methods).

**Table 4:** Calibration and optimal classification threshold for the 18 top-tier methods.

Rank	Feature Selection	Classifier	AUC (95% Range Across 100 CV Iterations)	Brier Score	Cal. Slope	Cal. Intercept	Youden Threshold	Sens. at Threshold	Spec. at Threshold
1	LASSO	Naive Bayes	0.959 (0.905–0.992)	0.082	1.02	−0.11	0.45	0.908	0.870
2	LASSO	Logistic Regression	0.957 (0.887–1.000)	0.080	0.91	0.01	0.54	0.885	0.905
3	LASSO	SVM	0.955 (0.895–0.992)	0.080	1.00	−0.01	0.57	0.883	0.906
4	RF importance	Logistic Regression	0.953 (0.879–0.997)	0.083	0.90	0.01	0.48	0.895	0.894
5	RFE	SVM	0.953 (0.876–0.995)	0.082	1.00	0.01	0.43	0.917	0.870
6	RF importance	Naive Bayes	0.953 (0.882–0.993)	0.089	0.91	−0.09	0.56	0.854	0.897
7	Boruta	SVM	0.952 (0.880–0.994)	0.083	1.00	0.00	0.50	0.899	0.890
8	RF importance	SVM	0.952 (0.879–0.996)	0.085	0.98	0.00	0.53	0.887	0.895
9	RFE	XGBoost	0.952 (0.883–0.995)	0.088	0.97	0.04	0.53	0.868	0.893
10	RFE	Random Forest	0.952 (0.889–0.994)	0.099	1.99	−0.02	0.53	0.877	0.890
11	LASSO	XGBoost	0.951 (0.887–0.991)	0.089	0.95	0.02	0.51	0.875	0.876
12	Boruta	XGBoost	0.951 (0.890–0.991)	0.089	0.97	0.05	0.56	0.854	0.902
13	ReliefF	Logistic Regression	0.951 (0.868–0.998)	0.085	0.90	0.00	0.44	0.899	0.880
14	Boruta	Random Forest	0.950 (0.890–0.993)	0.100	1.98	−0.02	0.55	0.854	0.906
15	RFE	Naive Bayes	0.950 (0.885–0.990)	0.091	0.79	−0.30	0.65	0.847	0.905
16	RFE	Logistic Regression	0.950 (0.870–0.999)	0.082	0.79	0.01	0.52	0.897	0.898
17	Correlation filter	Logistic Regression	0.950 (0.865–0.998)	0.085	0.90	0.00	0.49	0.887	0.894
18	RF importance	XGBoost	0.949 (0.872–0.994)	0.091	0.94	0.02	0.57	0.839	0.904

Calibration metrics were computed on pooled out-of-fold predicted probabilities across all 100 cross-validation folds, for the 18 methods classified as top-tier in Table 3 (Nemenyi post hoc *p* > 0.05 vs. the best method). Brier score = mean squared difference between predicted probability and observed outcome (lower is better; 0 = perfect). Calibration slope and intercept were estimated by logistic recalibration (observed outcome regressed on the logit of the predicted probability); a well-calibrated model has slope ≈ 1 and intercept ≈ 0. Youden threshold = probability cut-off maximizing sensitivity + specificity − 1 on the pooled predictions; sensitivity and specificity at this threshold are reported instead of the fixed 0.5 threshold used in Table 3.

**Table 5:** Stability of the six feature selection methods across 100 cross-validation folds.

Feature Selection Method	Mean Features (Range)	Nogueira Stability	Mean Jaccard	Core Features (100/100)	Mean Runtime (s)
LASSO	9.0 (8–10)	0.985	0.986	8	0.029
RF importance	9.0 (9–9)	0.907	0.917	7	0.237
Correlation filter	9.0 (9–9)	0.851	0.867	6	0.001
ReliefF	9.0 (9–9)	0.850	0.867	6	0.052
Boruta	14.6 (12–17)	0.594	0.859	11	8.656
RFE	15.7 (6–18)	0.171	0.802	6	2.380

Mean features (range) = mean (minimum–maximum) number of morphological parameters selected per fold. Nogueira stability index [21] ranges from 0 (no better than random selection) to 1 (perfectly stable), accounting for the number of features selected. Mean Jaccard = average pairwise Jaccard similarity between the feature subsets selected across the 100 folds. Core features = number of parameters selected in all 100/100 folds. Mean runtime = mean wall-clock computation time per fold, in seconds.

## Data Availability

The entire deidentified dataset, data dictionary, and analytic code for this investigation are available from the corresponding author upon reasonable request after publication by contacting Murat Onder at muratonder89@gmail.com.

## Supplementary Materials

The following supporting information can be downloaded at https://www.mdpi.com/article/10.3390/jcm15187246/s1. Supplementary Table S1. Definitions, imaging planes, anatomical landmarks, and formulas for the 18 MRI-based morphometric parameters used in this study. Supplementary Table S2. Interobserver and intraobserver reliability (intraclass correlation coefficients) of the 18 MRI-based morphometric measurements. Supplementary Table S3. Age- and sex-adjusted sensitivity analysis of the 18 morphometric parameters compared between the ACL-injury and control groups. Supplementary Table S4. Discrimination performance of all 36 feature selection × classifier combinations, with 95% confidence intervals for all metrics. Supplementary Figure S1. Representative MRI measurements of the five most influential morphometric parameters.

## Abbreviations

The following abbreviations are used in this manuscript:

ACL	Anterior cruciate ligament
AP	Anteroposterior
AUC	Area under the receiver operating characteristic curve
CI	Confidence interval
FDR	False discovery rate
FS	Feature selection
kNN	k-nearest neighbors
LASSO	Least absolute shrinkage and selection operator
LFCR	Lateral femoral condyle ratio
LTS	Lateral tibial slope
MCC	Matthews correlation coefficient
MRI	Magnetic resonance imaging
MTS	Medial tibial slope
NPV	Negative predictive value
NSI	Notch shape index
NWI	Notch width index
PACS	Picture archiving and communication system
PPV	Positive predictive value
RF	Random forest
RFE	Recursive feature elimination
SD	Standard deviation
SVM	Support vector machine
XGBoost	Extreme gradient boosting
