# Supplementary material for: MRI-Based Knee Morphometry and Pediatric Anterior Cruciate Ligament Injury: A Machine Learning Case–Control Study
Source: J Clin Med. 2026 Sep 17;15(18):7246. doi: 10.3390/jcm15187246 (PMC13607225; doi:10.3390/jcm15187246)
Supplement: Supplementary file 1 [file jcm-15-07246-s001.zip › jcm-4526861-supplementary/Supplementary_Table_S1.pdf]

Supplementary Table S1. Definitions, imaging planes, anatomical landmarks, and formulas for the 18 MRI-based morphometric parameters used in this study.

Parameter	MRI Plane	Anatomical Landmarks / Definition	Formula	Unit
Intercondylar notch width	Coronal	Distance between the medial and lateral walls of the intercondylar notch, measured at the standardized notch level.	Direct linear measurement	mm
Notch width index (NWI)	Coronal	Notch width and bicondylar femoral width measured at the same coronal level.	NWI = notch width / bicondylar femoral width	dimensionless
Lateral tibial slope (LTS)	Sagittal	Angle between a line perpendicular to the tibial longitudinal axis and a tangent to the lateral tibial plateau articular surface.	Direct angular measurement	degrees
Medial tibial slope (MTS)	Sagittal	Angle between a line perpendicular to the tibial longitudinal axis and a tangent to the medial tibial plateau articular surface.	Direct angular measurement	degrees
Medial meniscus–cartilage angle	Coronal	Angle between a line tangent to the superior surface of the medial meniscus and a line tangent to the medial tibial articular cartilage surface at the same level, on the central slice best showing the medial meniscal body.	Direct angular measurement	degrees
Medial tibial depth	Sagittal	Maximum perpendicular distance between a line connecting the anterior and posterior margins of the medial tibial articular surface and the deepest point of the medial plateau.	Direct linear measurement	mm
Lateral femoral condyle AP distance	Sagittal	Maximum anteroposterior dimension of the lateral femoral condyle (line A–B; see LFCR row).	Direct linear measurement	mm
Proximal tibial AP distance	Sagittal	Maximum anteroposterior dimension of the proximal tibial plateau.	Direct linear measurement	mm
Tibial eminence width	Coronal	Maximum transverse distance between the outer margins of the medial and lateral intercondylar eminences.	Direct linear measurement	mm
Tibial eminence width index (EWI)	Coronal	Tibial eminence width and tibial plateau width (maximum distance between the outer cortical margins of the medial and lateral tibial plateaus) measured on the same coronal image.	EWI = tibial eminence width / tibial plateau width	dimensionless
Notch-to-eminence ratio	Coronal	Intercondylar notch width and tibial eminence width measured on corresponding coronal images.	Notch width / eminence width	dimensionless
Notch shape index (NSI)	Coronal	Intercondylar notch width and notch height measured on corresponding coronal images.	NSI = notch width / notch height	dimensionless
Notch height	Coronal	Distance from the superior aspect of the intercondylar notch to the reference line at the inferior extent of the femoral condyles.	Direct linear measurement	mm
Lateral femoral condyle ratio (LFCR)	Sagittal	Distal femoral longitudinal axis defined by two best-fit circles fitted to the distal femoral shaft; midsagittal slice of the lateral condyle selected using the popliteal groove as a coronal reference; A = most anterior point and B = most posterior point of the lateral condyle on this slice; O = intersection of the femoral axis with line A–B.	LFCR = OB / AB	dimensionless
Coronal intercondylar notch angle	Coronal	Angle between lines drawn along the medial and lateral walls of the intercondylar notch.	Direct angular measurement	degrees
Axial intercondylar notch angle	Axial	Angle between lines drawn along the medial and lateral walls of the intercondylar notch, on the axial image best demonstrating the intercondylar region and posterior femoral condyles.	Direct angular measurement	degrees
Axial lateral wall angle	Axial	Angle between a posterior condylar reference line (connecting the most posterior cortical points of the femoral condyles) and a line tangent to the linear portion of the lateral intercondylar notch wall.	Direct angular measurement	degrees
Coronal lateral wall angle	Coronal	Angle between a distal femoral condylar reference line (connecting the most inferior articular points of the medial and lateral femoral condyles) and a line tangent to the lateral intercondylar notch wall.	Direct angular measurement	degrees

All measurements were performed by two independent observers using predefined anatomical landmarks; the mean of the two observers' measurements was used in subsequent analyses (see Section 2.5). AP = anteroposterior; NWI = notch width index; EWI = tibial eminence width index; NSI = notch shape index; LFCR = lateral femoral condyle ratio.
