# Supplementary material for: MRI-Based Knee Morphometry and Pediatric Anterior Cruciate Ligament Injury: A Machine Learning Case–Control Study
Source: J Clin Med. 2026 Sep 17;15(18):7246. doi: 10.3390/jcm15187246 (PMC13607225; doi:10.3390/jcm15187246)
Supplement: Supplementary file 1 [file jcm-15-07246-s001.zip › jcm-4526861-supplementary/Supplementary_Table_S2.pdf]

Supplementary Table S2. Interobserver and intraobserver reliability (intraclass correlation coefficients) of the 18 MRI-based morphometric measurements.

Parameter	N	Interobserver ICC (95% CI)	Intraobserver ICC – Observer 1 (95% CI)	Intraobserver ICC – Observer 2 (95% CI)
Lateral condyle AP distance (mm)	462	0.97 (0.964–0.975)	0.991 (0.989–0.993)	0.990 (0.988–0.991)
Notch width (mm)	462	0.96 (0.952–0.967)	0.991 (0.989–0.992)	0.989 (0.987–0.991)
Medial tibial depth (mm)	462	0.96 (0.952–0.966)	0.990 (0.988–0.992)	0.989 (0.986–0.990)
Proximal tibial AP distance (mm)	462	0.96 (0.952–0.967)	0.991 (0.989–0.992)	0.990 (0.988–0.991)
Intercondylar eminence width (mm)	462	0.96 (0.952–0.966)	0.990 (0.987–0.991)	0.989 (0.987–0.991)
Notch height (mm)	462	0.96 (0.952–0.967)	0.991 (0.989–0.992)	0.990 (0.988–0.992)
Notch width index	462	0.95 (0.940–0.958)	0.990 (0.988–0.991)	0.990 (0.989–0.992)
Intercondylar eminence width index	462	0.95 (0.941–0.958)	0.987 (0.984–0.989)	0.986 (0.983–0.988)
Lateral femoral condyle ratio	462	0.95 (0.940–0.958)	0.988 (0.985–0.990)	0.989 (0.986–0.991)
Notch/eminence ratio	462	0.94 (0.928–0.950)	0.990 (0.988–0.992)	0.989 (0.987–0.991)
Notch shape index	462	0.94 (0.928–0.950)	0.990 (0.988–0.992)	0.989 (0.987–0.991)
Lateral tibial slope (°)	462	0.93 (0.917–0.941)	0.982 (0.979–0.985)	0.984 (0.981–0.987)
Medial tibial slope (°)	462	0.93 (0.917–0.942)	0.983 (0.980–0.986)	0.981 (0.977–0.984)
Coronal intercondylar notch angle (°)	462	0.93 (0.916–0.941)	0.982 (0.978–0.985)	0.983 (0.979–0.986)
Axial intercondylar notch angle (°)	462	0.93 (0.917–0.941)	0.983 (0.980–0.986)	0.984 (0.980–0.986)
Medial meniscal cartilage angle (°)	462	0.92 (0.905–0.933)	0.983 (0.980–0.986)	0.981 (0.978–0.985)
Axial lateral wall angle (°)	462	0.92 (0.905–0.933)	0.979 (0.975–0.983)	0.982 (0.978–0.985)
Coronal lateral wall angle (°)	462	0.92 (0.905–0.933)	0.979 (0.975–0.983)	0.984 (0.980–0.986)

Interobserver reliability was assessed between the two independent observers on their first measurement of each parameter (n=462). Intraobserver reliability was assessed separately for each observer, comparing their first and second measurements of the same parameter, repeated after an interval and blinded to the original values. All intraclass correlation coefficients (ICC) were calculated using a two-way random-effects, absolute-agreement, single-measurement model. ICC = intraclass correlation coefficient; CI = confidence interval.
