# Supplementary material for: MRI-Based Knee Morphometry and Pediatric Anterior Cruciate Ligament Injury: A Machine Learning Case–Control Study
Source: J Clin Med. 2026 Sep 17;15(18):7246. doi: 10.3390/jcm15187246 (PMC13607225; doi:10.3390/jcm15187246)
Supplement: Supplementary file 1 [file jcm-15-07246-s001.zip › jcm-4526861-supplementary/Supplementary_Table_S3.pdf]

Supplementary Table S3. Age- and sex-adjusted sensitivity analysis of the 18 morphometric parameters compared between the ACL-injury and control groups.

Parameter	Unadjusted test	Unadjusted p (FDR)	Age/sex-adjusted p (FDR)	Rank-based sensitivity p (FDR)	Significant after adjustment
Axial intercondylar notch angle	Welch t-test	<0.001	<0.001	<0.001	Yes (retained)
Lateral femoral condyle ratio (LFCR)	Mann-Whitney U	<0.001	<0.001	<0.001	Yes (retained)
Medial tibial depth	Mann-Whitney U	<0.001	<0.001	<0.001	Yes (retained)
Medial tibial slope (MTS)	Mann-Whitney U	<0.001	<0.001	<0.001	Yes (retained)
Lateral tibial slope (LTS)	Mann-Whitney U	<0.001	<0.001	<0.001	Yes (retained)
Tibial eminence width	Mann-Whitney U	<0.001	<0.001	<0.001	Yes (retained)
Coronal intercondylar notch angle	Welch t-test	<0.001	<0.001	<0.001	Yes (retained)
Tibial eminence width index (EWI)	Mann-Whitney U	<0.001	<0.001	<0.001	Yes (retained)
Axial lateral wall angle	Mann-Whitney U	<0.001	<0.001	<0.001	Yes (retained)
Medial meniscus–cartilage angle	Mann-Whitney U	<0.001	<0.001	<0.001	Yes (retained)
Notch shape index (NSI)	Mann-Whitney U	0.003	0.038	0.005	Yes (retained)
Lateral femoral condyle AP distance	Mann-Whitney U	0.008	0.005	0.004	Yes (retained)
Notch width index (NWI)	Mann-Whitney U	0.021	0.032	0.025	Yes (retained)
Coronal lateral wall angle	Welch t-test	0.054	0.076	0.088	—
Intercondylar notch width	Mann-Whitney U	0.085	0.324	0.089	—
Notch-to-eminence ratio	Mann-Whitney U	0.099	0.064	0.088	—
Notch height	Mann-Whitney U	0.607	0.670	0.756	—
Proximal tibial AP distance	Mann-Whitney U	0.932	0.571	0.903	—

Unadjusted test selected per parameter according to the Shapiro-Wilk normality result (Welch t-test if normal, Mann-Whitney U otherwise), consistent with Table 2. Adjusted analysis: linear regression of each parameter on group, age, and sex (ANCOVA); rank-based sensitivity analysis: identical model fitted to rank-transformed parameter values, for parameters not meeting normality assumptions. All p-values are Benjamini-Hochberg FDR-corrected across the 18 parameters, calculated separately within each of the three columns. Skeletal maturity (e.g., Tanner stage, bone age) was not recorded in this cohort and could not be included in the adjustment.
