# Supplementary material for: MRI-Based Knee Morphometry and Pediatric Anterior Cruciate Ligament Injury: A Machine Learning Case–Control Study
Source: J Clin Med. 2026 Sep 17;15(18):7246. doi: 10.3390/jcm15187246 (PMC13607225; doi:10.3390/jcm15187246)
Supplement: Supplementary file 1 [file jcm-15-07246-s001.zip › jcm-4526861-supplementary/Supplementary_Table_S4.pdf]

Supplementary Table S4. Discrimination performance of all 36 feature selection × classifier combinations, with 95% confidence intervals for all metrics.

Rank	Feature selection	Classifier	AUC (95% Range)	Accuracy (95% Range)	Sensitivity (95% Range)	Specificity (95% Range)	PPV (95% Range)	NPV (95% Range)	F1 (95% Range)	MCC (95% Range)	Top-tier
1	LASSO	Naive Bayes	0.959 (0.905–0.992)	0.883 (0.765–0.957)	0.887 (0.783–0.980)	0.879 (0.739–1.000)	0.885 (0.749–1.000)	0.887 (0.791–0.979)	0.885 (0.784–0.957)	0.769 (0.533–0.916)	Yes
2	LASSO	Logistic Regression	0.957 (0.887–1.000)	0.893 (0.793–0.957)	0.891 (0.739–1.000)	0.895 (0.739–1.000)	0.899 (0.769–1.000)	0.894 (0.769–1.000)	0.893 (0.800–0.959)	0.789 (0.588–0.918)	Yes
3	LASSO	SVM	0.955 (0.895–0.992)	0.892 (0.804–0.968)	0.898 (0.760–1.000)	0.886 (0.739–1.000)	0.891 (0.773–1.000)	0.898 (0.774–1.000)	0.893 (0.800–0.970)	0.787 (0.608–0.939)	Yes
4	RF importance	Logistic Regression	0.953 (0.879–0.997)	0.892 (0.793–0.978)	0.887 (0.739–1.000)	0.896 (0.777–1.000)	0.900 (0.775–1.000)	0.890 (0.769–1.000)	0.892 (0.795–0.979)	0.787 (0.586–0.957)	Yes
5	RFE	SVM	0.953 (0.876–0.995)	0.892 (0.791–0.968)	0.899 (0.760–1.000)	0.885 (0.739–1.000)	0.891 (0.773–1.000)	0.899 (0.783–1.000)	0.893 (0.800–0.970)	0.787 (0.582–0.939)	Yes
6	RF importance	Naive Bayes	0.953 (0.882–0.993)	0.872 (0.763–0.968)	0.873 (0.739–1.000)	0.872 (0.693–1.000)	0.878 (0.741–1.000)	0.873 (0.745–1.000)	0.873 (0.759–0.969)	0.748 (0.529–0.938)	Yes
7	Boruta	SVM	0.952 (0.880–0.994)	0.895 (0.785–0.978)	0.899 (0.760–1.000)	0.890 (0.760–1.000)	0.896 (0.783–1.000)	0.899 (0.783–1.000)	0.896 (0.787–0.978)	0.792 (0.570–0.957)	Yes
8	RF importance	SVM	0.952 (0.879–0.996)	0.890 (0.804–0.957)	0.893 (0.739–1.000)	0.887 (0.755–1.000)	0.892 (0.780–1.000)	0.894 (0.769–1.000)	0.891 (0.795–0.958)	0.783 (0.608–0.917)	Yes
9	RFE	XGBoost	0.952 (0.883–0.995)	0.878 (0.766–0.957)	0.875 (0.739–0.980)	0.882 (0.733–0.979)	0.885 (0.750–0.980)	0.877 (0.738–0.980)	0.878 (0.758–0.956)	0.759 (0.539–0.914)	Yes
10	RFE	Random Forest	0.952 (0.889–0.994)	0.879 (0.783–0.968)	0.890 (0.750–1.000)	0.868 (0.716–1.000)	0.877 (0.750–1.000)	0.888 (0.776–1.000)	0.882 (0.787–0.969)	0.762 (0.571–0.939)	Yes
11	LASSO	XGBoost	0.951 (0.887–0.991)	0.874 (0.768–0.947)	0.876 (0.744–1.000)	0.872 (0.733–1.000)	0.878 (0.759–1.000)	0.877 (0.747–1.000)	0.875 (0.762–0.948)	0.752 (0.540–0.894)	Yes
12	Boruta	XGBoost	0.951 (0.890–0.991)	0.877 (0.760–0.946)	0.873 (0.702–1.000)	0.880 (0.739–1.000)	0.884 (0.761–1.000)	0.876 (0.729–1.000)	0.877 (0.747–0.949)	0.756 (0.531–0.897)	Yes
13	ReliefF	Logistic Regression	0.951 (0.868–0.998)	0.888 (0.787–0.968)	0.884 (0.723–1.000)	0.893 (0.755–1.000)	0.897 (0.778–1.000)	0.887 (0.745–1.000)	0.888 (0.776–0.970)	0.780 (0.581–0.939)	Yes
14	Boruta	Random Forest	0.950 (0.890–0.993)	0.876 (0.783–0.968)	0.885 (0.744–1.000)	0.867 (0.716–0.957)	0.875 (0.754–0.959)	0.885 (0.769–1.000)	0.878 (0.786–0.969)	0.756 (0.569–0.938)	Yes
15	RFE	Naive Bayes	0.950 (0.885–0.990)	0.869 (0.761–0.936)	0.889 (0.760–1.000)	0.850 (0.673–0.957)	0.862 (0.727–0.956)	0.886 (0.775–1.000)	0.873 (0.760–0.939)	0.743 (0.524–0.877)	Yes
16	RFE	Logistic Regression	0.950 (0.870–0.999)	0.897 (0.771–0.968)	0.898 (0.739–1.000)	0.895 (0.777–1.000)	0.900 (0.765–1.000)	0.900 (0.761–1.000)	0.897 (0.774–0.970)	0.797 (0.543–0.939)	Yes
17	Correlation filter	Logistic Regression	0.950 (0.865–0.998)	0.890 (0.795–0.968)	0.885 (0.744–1.000)	0.894 (0.755–1.000)	0.897 (0.778–1.000)	0.888 (0.755–1.000)	0.890 (0.781–0.970)	0.782 (0.592–0.939)	Yes
18	RF importance	XGBoost	0.949 (0.872–0.994)	0.867 (0.758–0.957)	0.864 (0.716–0.980)	0.870 (0.716–1.000)	0.875 (0.741–1.000)	0.867 (0.731–0.980)	0.868 (0.753–0.957)	0.738 (0.519–0.914)	Yes
19	LASSO	Random Forest	0.949 (0.883–0.994)	0.869 (0.763–0.957)	0.877 (0.739–1.000)	0.862 (0.696–1.000)	0.869 (0.736–1.000)	0.876 (0.750–1.000)	0.871 (0.760–0.957)	0.742 (0.529–0.916)	No
20	Boruta	Logistic Regression	0.949 (0.866–0.995)	0.894 (0.795–0.957)	0.893 (0.760–1.000)	0.895 (0.783–1.000)	0.899 (0.796–1.000)	0.895 (0.779–1.000)	0.894 (0.800–0.958)	0.791 (0.590–0.917)	No
21	ReliefF	Naive Bayes	0.949 (0.882–0.993)	0.865 (0.761–0.957)	0.871 (0.723–1.000)	0.858 (0.709–0.979)	0.866 (0.745–0.980)	0.871 (0.757–1.000)	0.866 (0.763–0.957)	0.733 (0.522–0.913)	No
22	Correlation filter	SVM	0.948 (0.868–0.995)	0.887 (0.772–0.957)	0.892 (0.739–1.000)	0.881 (0.733–1.000)	0.888 (0.754–1.000)	0.892 (0.750–1.000)	0.888 (0.776–0.958)	0.776 (0.546–0.917)	No
23	ReliefF	SVM	0.948 (0.879–0.995)	0.891 (0.780–0.968)	0.898 (0.739–1.000)	0.883 (0.739–0.979)	0.890 (0.763–0.981)	0.899 (0.750–1.000)	0.892 (0.776–0.969)	0.785 (0.571–0.939)	No
24	Correlation filter	XGBoost	0.947 (0.886–0.995)	0.864 (0.742–0.957)	0.858 (0.680–0.958)	0.870 (0.739–1.000)	0.874 (0.756–1.000)	0.862 (0.722–0.957)	0.864 (0.738–0.956)	0.732 (0.484–0.916)	No
25	Boruta	Naive Bayes	0.947 (0.884–0.984)	0.858 (0.742–0.936)	0.877 (0.744–0.958)	0.838 (0.696–0.957)	0.850 (0.731–0.955)	0.874 (0.751–0.955)	0.861 (0.761–0.936)	0.719 (0.492–0.872)	No
26	RF importance	Random Forest	0.946 (0.882–0.992)	0.869 (0.761–0.957)	0.872 (0.739–1.000)	0.866 (0.673–1.000)	0.873 (0.736–1.000)	0.873 (0.750–1.000)	0.870 (0.769–0.957)	0.742 (0.528–0.916)	No
27	ReliefF	XGBoost	0.946 (0.882–0.996)	0.866 (0.746–0.978)	0.857 (0.702–0.958)	0.874 (0.696–1.000)	0.878 (0.725–1.000)	0.861 (0.729–0.958)	0.865 (0.739–0.978)	0.735 (0.500–0.957)	No
28	Correlation filter	Naive Bayes	0.945 (0.884–0.985)	0.864 (0.761–0.957)	0.882 (0.750–0.980)	0.845 (0.673–0.957)	0.857 (0.736–0.958)	0.879 (0.763–0.979)	0.867 (0.783–0.957)	0.732 (0.532–0.914)	No
29	Correlation filter	Random Forest	0.944 (0.872–0.992)	0.866 (0.761–0.947)	0.875 (0.739–1.000)	0.856 (0.696–1.000)	0.864 (0.741–1.000)	0.874 (0.750–1.000)	0.868 (0.764–0.948)	0.735 (0.529–0.899)	No
30	ReliefF	Random Forest	0.944 (0.868–0.992)	0.871 (0.761–0.957)	0.879 (0.716–1.000)	0.863 (0.716–0.979)	0.871 (0.745–0.981)	0.879 (0.745–1.000)	0.873 (0.762–0.957)	0.746 (0.528–0.917)	No
31	RF importance	kNN	0.926 (0.857–0.986)	0.855 (0.747–0.936)	0.813 (0.638–0.957)	0.899 (0.800–0.979)	0.893 (0.786–0.980)	0.830 (0.701–0.956)	0.848 (0.716–0.940)	0.717 (0.506–0.875)	No
32	ReliefF	kNN	0.923 (0.829–0.988)	0.856 (0.742–0.936)	0.810 (0.652–0.957)	0.902 (0.783–1.000)	0.897 (0.773–1.000)	0.828 (0.714–0.951)	0.848 (0.739–0.936)	0.718 (0.494–0.875)	No
33	Correlation filter	kNN	0.920 (0.825–0.984)	0.863 (0.750–0.947)	0.830 (0.696–0.957)	0.896 (0.773–1.000)	0.893 (0.767–1.000)	0.842 (0.720–0.955)	0.858 (0.742–0.947)	0.731 (0.504–0.897)	No
34	RFE	kNN	0.915 (0.815–0.978)	0.817 (0.709–0.935)	0.726 (0.565–0.915)	0.909 (0.783–1.000)	0.894 (0.750–1.000)	0.770 (0.667–0.917)	0.797 (0.674–0.932)	0.649 (0.427–0.872)	No
35	Boruta	kNN	0.914 (0.800–0.975)	0.823 (0.725–0.924)	0.738 (0.583–0.913)	0.910 (0.777–1.000)	0.895 (0.767–1.000)	0.778 (0.677–0.917)	0.806 (0.692–0.924)	0.660 (0.460–0.855)	No
36	LASSO	kNN	0.908 (0.824–0.981)	0.858 (0.761–0.936)	0.810 (0.652–0.938)	0.906 (0.739–1.000)	0.900 (0.764–1.000)	0.827 (0.717–0.935)	0.851 (0.735–0.936)	0.722 (0.522–0.873)	No

Results from repeated 10-fold cross-validation (10 repeats, 100 folds total); feature selection was performed independently within each training fold to prevent data leakage. All metrics except AUC were computed at a fixed probability threshold of 0.5. Values are mean (95% range across 100 CV iterations, 2.5th–97.5th percentile); because folds are not statistically independent, this range should not be interpreted as a conventional confidence interval for the population mean. PPV = positive predictive value; NPV = negative predictive value; MCC = Matthews correlation coefficient. Top-tier = not statistically significantly different from the best-ranked method (Nemenyi post-hoc $p>0.05$, following a significant Friedman test: $\chi^2=894.2$, df=35, $p<0.001$; n=18 of 36 methods). This table corresponds to Table 3 in the main text, reported here with confidence intervals for all discrimination metrics rather than AUC alone.
